# Autophagy and Bacterial infections

**DOI:** 10.1080/27694127.2025.2542904

**Published:** 2025-09-01

**Authors:** Ken Cadwell, Clara Abraham, Shai Bel, Santosh Chauhan, Jörn Coers, María I. Colombo, Jacob R Davis, Daniel Hofius, Hang Thi Thu Nguyen, Michinaga Ogawa, Craig R. Roy, Feng Shao, Sayaka Shizukuishi, Christina L. Stallings, Magdalena Szczesna, Gergory Taylor, Teresa LM Thurston, Robert Watson, Thomas Wileman, Yue Xu, Dario S. Zamboni

**Affiliations:** a Division of Gastroenterology and Hepatology, Department of Medicine, University of Pennsylvania Perelman School of Medicine, Philadelphia, PA, USA; b Department of Pathobiology, University of Pennsylvania School of Veterinary Medicine, Philadelphia, PA, USA; c Department of Internal Medicine, Yale University, New Haven, CT, USA; d Azrieli Faculty of Medicine, Bar-ilan University, Safed, Israel; e Cell Biology and Infectious Diseases Unit, CSIR-Centre for Cellular and Molecular Biology, Hyderabad, India; f Departments of Molecular Genetics and Microbiology and Integrative Immunobiology, Duke University Medical School, Durham, NC, USA; g School of Medicine, Laboratory of molecular mechanisms involved in vesicular trafficking and autophagy, IHEM-CONICET- Universidad Nacional de Cuyo, Mendoza, Argentina; h Department of Microbial Pathogenesis and Immunology, Texas A&M Health, College of Medicine, Bryan, TX, USA; i Department of Plant Biology, Uppsala BioCenter, Swedish University of Agricultural Sciences (SLU), Uppsala, Sweden; j Linnean Center for Plant Biology, Uppsala, Sweden; k M2iSH (Microbes, intestine, inflammation and Susceptibility of the Host), U1071 Inserm, University of Clermont Auvergne, INRAE USC 1382, Clermont-Ferrand, France; l Department of Bacteriology I, National Institute of Infectious Diseases, JIHS, Tokyo, Japan; m Department of Microbial Pathogenesis, School of Medicine, Yale University, New Haven, CT, USA; n National Institute of Biological Sciences, Beijing, China; o Department of Molecular Microbiology, Center for Women’s Infectious Disease Research, Washington University School of Medicine, St. Louis, MO, USA; p Department of Infectious Disease, Centre for Bacterial Resistance Biology, Imperial College London, London, UK; q Departments of Medicine, Molecular Genetics and Microbiology, and the Center for the Study of Aging and Human Development, Duke University Medical Center, Durham, NC, USA; r Geriatrics Research and Clinical Center, Durham VA Health Care Center, Durham, NC, USA; s Sir William Dunn School of Pathology, University of Oxford, Oxford, UK; t Department of Medicine, Division of Infectious Diseases, Vanderbilt University Medical Center, Nashville, TN, USA; u Biomedical Research Centre, Norwich Medical School, University of East Anglia, Norwich, Norfolk, UK; v Department of Pathophysiology, Shanghai Jiao Tong University School of Medicine, Shanghai, China; w Department of Cell Biology, School of Medicine of Ribeirão Preto, University of São Paulo, São Paulo, Brazil

**Keywords:** Autophagy, bacteria, xenophagy, CASM, LC3 associated phagocytosis, Crohn disease

## Abstract

Autophagy is an evolutionarily conserved cellular process that is prominent during bacterial infections. In this review article, we discuss how direct pathogen clearance via xenophagy and regulation of inflammatory products represent dual functions of autophagy that coordinate an effective antimicrobial response. We detail the molecular mechanisms of xenophagy, including signals that indicate the presence of an intracellular pathogen and autophagy receptor-mediated cargo targeting, while highlighting pathogen counterstrategies, such as bacterial effector proteins that inhibit autophagy initiation or exploit autophagic membranes for replication. Pathways that are related to autophagy, including LC3-associated phagocytosis (LAP) and conjugation of ATG8 to single membranes (CASM), are expanding the role of autophagy in antimicrobial defense beyond traditional double-membrane autophagosomes. Examination of Crohn disease-associated genes links impaired autophagy to inflammation and defective bacterial handling. We propose emerging concepts, such as effector-triggered immunity, where autophagy inhibition by pathogens triggers inflammatory defenses and discusses the therapeutic potential of modulating autophagy in infectious and inflammatory diseases.

## Introduction

### Autophagy is mediated by a series of protein complexes

Autophagy is an evolutionarily conserved process found in all eukaryotic cells, from single-celled yeast to complex multicellular organisms like humans. Although its fundamental role in recycling cellular components during starvation has been preserved throughout evolution, multicellular organisms have expanded the functions of autophagy to include specialized roles in immunity, development, and tissue homeostasis [1]. This ancient pathway operates both constitutively to maintain cellular quality control and can be strongly induced in response to stress signals such as nutrient deprivation, infection, or organelle damage.

The term autophagy is used interchangeably with macroautophagy and involves sequestration of cytosolic material within a double-membrane vesicle called the autophagosome that delivers this cargo to the lysosome, distinguishing this process from other pathways such as chaperone-mediated autophagy or microphagy in which cytosolic cargo is transported directly to the lysosome. The molecular machinery of autophagy is organized into functional complexes of ATG (autophagy related) proteins that act sequentially to drive autophagosome formation [2]. The process begins with activation of the pre-initiation complex, consisting of the ULK1 kinase (or its homolog ULK2), ATG13, RB1CC1/FIP200, and ATG101. This complex integrates upstream signals from nutrient sensors. For instance, when active under nutrient-rich conditions, MTORC1 phosphorylates and inhibits ULK1; during starvation, MTORC1 inhibition allows AMPK to activate ULK1 through phosphorylation at Ser317 and Ser777. The activated ULK1 complex translocates to endoplasmic reticulum (ER)-associated sites called omegasomes, named after their structural likeness to the Greek symbol omega. The ULK1 complex phosphorylates components of the downstream initiation complex, also known as the class III phosphatidylinositol 3 kinase (PtdIns3K) complex, composed of the lipid kinase PIK3C3/VPS34, its regulatory subunit PIK3R4/VPS15, the scaffolding protein BECN1/ATG6, and the autophagy-specific factor ATG14. This complex generates phosphatidylinositol-3-phosphate (PtdIns3P), creating a platform for recruitment of additional autophagy machinery necessary for membrane expansion including the WIPI (WD repeat domain, phosphoinositide interacting) proteins, the lipid transporter ATG2, and the transmembrane protein with lipid scramblase activity ATG9A.

The cup-shaped membrane structure visible at this step that begins to surround cellular cargo has been referred to as the isolation membrane or phagophore. The membrane source for this growing structure likely comes from multiple organelles including the ER, mitochondria-ER contact sites, the Golgi apparatus, and recycling endosomes [3–5]. ATG2 and ATG9A work together to transfer lipids to the phagophore and equilibrate them into the opposing leaflet [6,7]. Two ubiquitin-like conjugation systems with shared components facilitate membrane expansion and cargo recruitment. The first involves the covalent conjugation of the ubiquitin-like protein ATG12 to ATG5 through sequential action of the E1-like enzyme ATG7 and the E2-like enzyme ATG10. The ATG12–ATG5 conjugate then non-covalently associates with ATG16L1 to form an E3-like complex that localizes to the growing phagophore membrane through association with WIPI2B.[8,9] The second system mediates the lipidation of ubiquitin-like Atg8-family proteins, including MAP1LC3/LC3. The newly synthesized LC3 is first cleaved by ATG4 proteases to expose a C-terminal glycine, then conjugated to the lipid phosphatidylethanolamine through the coordinated action of ATG7 (E1), ATG3 (E2), and the ATG12–ATG5-ATG16L1 complex (E3). This lipidated form of LC3 becomes stably associated with both the inner and outer phagophore membranes and the population on the concave surface remains within the completed autophagosome.

Following autophagosome formation, vesicle trafficking proteins that overlap with other pathways mediate fusion with endosomes and multivesicular bodies, to form the amphisome, and lysosomal compartments, to form the autolysosome. The RAB GTPase RAB7 is central in this process, [10] recruiting effectors like the HOPS (homotypic fusion and protein sorting) complex that bridges autophagosomes with lysosomes. SNARE proteins including STX17 (syntaxin 17), SNAP29, and VAMP8 mediate membrane fusion, while UVRAG (UV radiation resistance associated) interacts with the class C VPS complex to promote autophagosome maturation. This entire process is highly dynamic, with regulatory inputs from nutrient status, energy sensors, and cellular stress pathways constantly modulating the activity of each component to maintain cellular homeostasis. Additional protein interactions and post-translational modifications mediate crosstalk between different parts of the process. For example, RB1CC1 and ATG16L1 directly interact to increase the efficiency by which the signals provided by the pre-initiation complex result in LC3 lipidation during nutrient deprivation [11,12].

### Autophagy cargo and specialized functions of the pathway

In addition to bulk degradation of cytosolic constituents, cargo can be selectively targeted to autophagosomes through specific recognition systems. Ubiquitinated targets, such as damaged mitochondria or invading bacteria, are recognized by autophagy receptors like SQSTM1/p62, CALCOCO2/NDP52, TAX1BP1/T6BP, and OPTN (optineurin). These receptors bridge the cargo to the phagophore membrane by binding both ubiquitin and LC3 proteins. Beyond acting as a bridge, CALCOCO2 and TAX1BP1 interact with Myosin VI to regulate autophagosome maturation, coupling cargo detection with degradation[13]. Other targeting mechanisms involve direct recognition of cargo by specialized receptors, for example BNIP3L/Nix for mitochondria or the danger receptor LGALS8 for damaged sterile or pathogen-containing vacuoles. LGALS8 then acts as a cargo adaptor to recruit autophagy receptors like CALCOCO2 and TAX1BP1 in a ubiquitin-independent manner [11]. Specific terms, such as mitophagy for mitochondria and xenophagy for pathogens, are used to describe cargo-specific autophagy [14].

The LC3 protein family, central to autophagosome formation, consists of multiple isoforms with distinct but overlapping functions. In mammals, there are six Atg8 homologs divided into two subfamilies: the LC3 subfamily (LC3A, LC3B, LC3C) and the GABARAP subfamily (GABARAP, GABARAPL1, GABARAPL2). Although all family members participate in autophagosome formation and function, they show preferences for different types or steps of autophagy. For example, LC3C is particularly important for xenophagy while GABARAP proteins are more involved in autophagosome maturation and fusion with lysosomes [15,16]. Atg8 homologs can also associate with single membranes in autophagy-related processes.

In a cell type-dependent manner, autophagy under basal conditions removes long-lived proteins and damaged organelles. These functions are critical for preventing age-related degeneration in neurons, offsetting the high secretory burden of intestinal Paneth cells, and many other physiological settings. When induced by stress signals, autophagy activity can increase dramatically. Nutrient starvation remains the best-characterized inducer, acting through the MTORC1 kinase which integrates signals from growth factors, amino acids, and cellular energy status. Other inducers include hypoxia through HIF1A/HIF-1α, oxidative stress via NFE2L2/NRF2 activation, protein misfolding, and infection through pattern recognition receptors (PRRs). A recurring theme is the crosstalk between autophagy and PRRs such as toll-like receptors (TLRs), which are membrane bound sensors that signal through the adaptor MYD88 and TICAM1/TRIF in the presence of microbial ligands (e.g., nucleic acids and bacterial cell wall) in the endosome and at the plasma membrane, and NOD-like receptors (NLRs), which are activated by microbial products in the cytosol.

In multicellular organisms, autophagy has acquired additional specialized functions beyond nutrient recycling. It contributes to development by remodeling tissues during metamorphosis in insects and embryonic development in mammals.[2] The immune system employs autophagy for direct pathogen elimination (xenophagy), antigen presentation, lymphocyte homeostasis and regulation of cytokines (soluble immune mediators) that are produced downstream of the PRRs mentioned above.[17] In particular, autophagy restrains inflammasomes, innate immune complexes composed of NLRs and caspases that activate cytokines IL1B/IL-1β (interleukin 1 beta) and IL18 and an inflammatory form of cell death termed pyroptosis. Autophagy also functions both upstream and downstream of interferons (IFNs), a class of cytokines that induce a suite of antiviral and antimicrobial effector proteins. In the gut, autophagy maintains the delicate balance with commensal bacteria by supporting the function of secretory intestinal epithelial cells like Paneth cells and goblet cells.

In this review, we will discuss the complex interplay between autophagy and bacterial infections. A common theme that bridges different sections of this article is that autophagy proteins regulate intracellular and extracellular interactions with microbes through interconnected membrane trafficking processes. These functions often appear contradictory, such as promoting self-defense while also dampening inflammation to avoid excess immune reactions. These functions can coexist because they are often separated by time and space, and they are mobilized in a pathogen- or context-specific manner. These functions can coexist because they are often separated by time and space, and they are mobilized in a pathogen- or context-specific manner. Xenophagy serves as a powerful cell-autonomous defense mechanism. However, host-microbe co-evolution has selected for pathogens with sophisticated countermeasures, producing effector proteins that block autophagy initiation, prevent autophagosome maturation, or even exploit autophagic membranes for replication. We will discuss the core autophagy machinery and its adaptations for pathogen recognition, communication with neighboring cells, and processes that are independent of autophagosomes. We will also discuss how defects in autophagy contribute to inflammatory diseases and therapeutic outlook.

## Mechanics of xenophagy

The term xenophagy arises from the prefix “xeno,” meaning “foreign” and refers to the targeted elimination of pathogens including bacteria, viruses and parasites. Although the induction of autophagosome formation upon pathogen exposure was observed as early as 1984, [18] it took many more years to delineate the molecular machinery required to initiate and regulate xenophagy.

Bacteria that enter host cells initially reside in a membrane limited pathogen-containing vacuole (PcV). Upon damage of this vacuole, bacteria become exposed to the cytosol and xenophagy is initiated. In this way, xenophagy helps maintain a sterile cytosolic environment, restricting the growth of several intracellular pathogens, including *Salmonella enterica* serovar Typhimurium, [19] *Mycobacterium tuberculosis* (*Mtb*), [20] and group A *streptococcus* (GAS)[21]. Since these early studies and the analysis of how intracellular pathogens evade sequestration by the phagophore, progress over the last two decades has delineated important features of xenophagy.

In this section, we focus on the mechanics of how xenophagy encloses bacteria into *de novo* double membrane autophagosomes following breakdown of the PcV. In general, xenophagy utilizes a hierarchy of ATG proteins in a manner similar to starvation-induced autophagy: (1) initiation and autophagosome nucleation, (2) membrane expansion and elongation, (3) autophagosome-lysosome fusion and (4) cargo degradation [22]. Yet, differences exist and uncovering the specifics of xenophagy initiation and control are essential to our understanding of this ancient form of immunity.

### Membrane rupture as a danger signal

Whereas bulk autophagy is induced by starvation- or stress-induced mTOR inhibition and is more inclusive of various cargo, xenophagy is highly selective and initiates specifically on the foreign cargo. Therefore, as with other forms of selective autophagy, such as mitophagy, xenophagy relies on eat me signals that label the cargo. A potential model that requires investigation is one in which each autophagy receptor can induce *in situ* autophagosome biogenesis, bypassing the need for upstream signals such that xenophagy can occur in infected cells without starvation.

**Figure 1.**
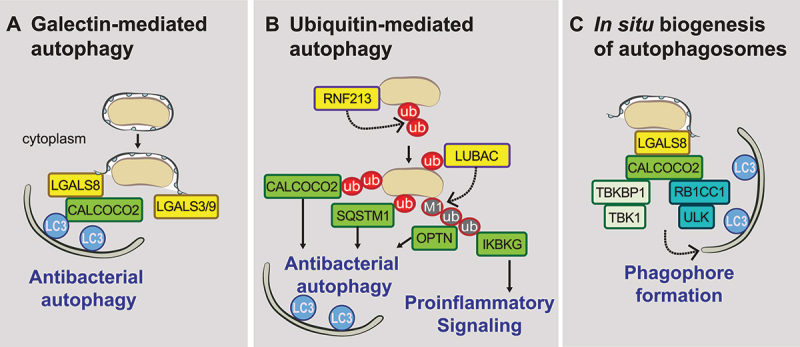
Molecular mechanisms of xenophagy. (A) Carbohydrates on the ruptured pathogen-containing vacuole act as eat-me signals to recruit members of the galectin-family of carbohydrate binding proteins. LGALS8 acts as a damage detecting receptor, interacting with CALCOCO2 to induce xenophagy. (B) Ubiquitin, deposited either directly on the lipopolysaccharide of bacteria like *Salmonella*, or on unknown substrates, initiates the recruitment of other E3 ligases, including linear ubiquitin chain assembly complex (LUBAC). This recruits autophagy receptors including CALCOCO2 (and TAX1BP1), SQSTM1 and OPTN (optineurin), as well as the proinflammatory signaling regulator, IKBKG/NEMO. (C) CALCOCO2, via its interactions with RB1CC1/FIP200-ULK and TBKBP1/SINTBAD-TBK1, initiates the *in situ* biogenesis of autophagosomes.

Although the precise entry mechanism of a bacterium into a host [23,24] cell varies, exit from the ensuing vacuole requires damage of this host-derived membrane. The detection of membrane rupture as a danger signal and initiator of xenophagy, therefore endows host cells with the capacity to sequester diverse pathogens. Galectins are a family of cytosolic carbohydrate binding proteins, several of which detect host-derived sugars following the rupture of PcVs [25,26]. This exposure of host glycans, such as β-galactosides normally present on the inner surface of vacuoles, triggers the recruitment of the autophagy machinery. In the case of *Salmonella*, a Gram-negative bacterium associated with food poisoning and gastroenteritis, rupture of the PcV induces the recruitment of LGALS3 (galectin 3) LGALS8 and LGALS9, with LGALS8 playing a unique role as a danger receptor that inhibits bacterial proliferation (Figure 1A) [26].

Other intracellular pathogens that cause foodborne diseases, such as *Shigella flexneri* and *Listeria monocytogenes*, also trigger the recruitment of several galectin-family members when vacuolar integrity is compromised [25,26]. For *Mtb*, the lung pathogen that causes tuberculosis, LGALS3 in concert with TRIM16 coordinates the mobilization of several autophagy regulators in response to damage of bacteria-containing endosomes [27], as does LGALS8[28]. LGALS1 and LGALS7 support xenophagy and restrict the replication of GAS, Gram-positive bacteria (*Streptococcus pyogenes*) that cause a range of diseases including strep throat [29]. Therefore, the cargo, the mechanism of vacuole rupture, or the precise membrane constituents might dictate which galectins initiate and regulate xenophagy induction.

### Ubiquitin as an eat-me signal

In addition to the galectin-dependent pathway of xenophagy, poly-ubiquitin chains, present on either the vacuole or as a “coat” surrounding the cytosol-exposed pathogen, [30] act as an important signal that labels the bacteria as cargo for destruction. Amplification and remodeling of the initial ubiquitin coat through the recruitment of the E3 ubiquitin ligase, LUBAC, results in the generation of linear (M1-linked) polyubiquitin patches. This not only drives xenophagy but also induces recruitment of the pro-inflammatory signaling adaptor IKBKG/NEMO [31,32]. In this way, the ubiquitin coat generates a signaling platform on the pathogen that results in a multi-layered immune response that encompasses different signaling cascades (Figure 1B). Several other E3-ligases are associated with the generation of different poly-ubiquitin chain types on the pathogen’s ubiquitin coat, including LRSAM1 [33], PRKN/parkin [34], SMURF1 [35], ARIH1 [36] and RNF166 [37]. Some of these, like PRKN, also regulate mitophagy, again highlighting similarities between these two pathways. Although the specific roles of each of these enzymes remain unclear, one hypothesis is that the generation of different chain linkages creates a robust and regulated anti-bacterial system [36].

The discovery that the E3 ligase RNF213 ubiquitinates the lipopolysaccharide (LPS) of *Salmonella* addressed the long-standing question as to how ubiquitin-coat formation is initiated (Figure 1B) [38]. RNF213, which is the largest mammalian E3 ligase, requires both its dynein-like domain for ATP hydrolysis and RZ domain to recognize, coat, and then ubiquitinate LPS (specific to Gram-negative bacteria) and other substrates associated with Gram-positive bacteria and the parasite *Toxoplasma gondii* [38,39]. Although the direct substrates beyond LPS are currently unknown, it is now evident that RNF213 has broad roles in the recognition and growth restriction of diverse intracellular pathogens [39–42]. Given that RNF213 is under strong positive selection [39], it is likely that this protein represents a new PRR of our innate immune system.

### Autophagy receptors as readers of eat-me signals

Given that selective autophagy targets many different cellular substrates, including mitochondria, ribosomes, lipid droplets, and protein aggregates for degradation, it is perhaps not surprising that over 30 different autophagy receptors have been identified [43]. For xenophagy, four key autophagy receptors, SQSTM1, CALCOCO2 and its homolog TAX1BP1, and OPTN, play a central role [44–46]. Of interest, these four autophagy receptors, together with NBR1, are also involved in mitophagy [47]. Given that mitochondria evolved following the engulfment of a *Rickettsia*-like α-protobacterium, it is possible that xenophagy and mitophagy diverged from a common form of selective autophagy that targeted invading microbes [48].

Although each of the above four autophagy receptors interact with ubiquitin, only CALCOCO2 and TAX1BP1 recognize a LGALS8-dependent eat-me signal, which further enhances their contribution to pathogen clearance (Figure 1A–B) [26,28]. Central to the function of all xenophagy receptors is their ability to interact with LC3/GABARAP via a short LC3-interacting region (LIR). The importance of the LIR is highlighted by mutational studies, as well as the observation that phosphorylation of OPTN by TBK1 (TANK binding kinase 1) enhances its LC3 binding affinity to promote pathogen clearance [45].

### In situ autophagosome biogenesis

During xenophagy of *Salmonella*, the autophagy receptor CALCOCO2 both detects the eat me signal and recruits the upstream ULK-RB1CC1 pre-initiation complex [49]. This *in situ* assembly of the upstream autophagy machinery results in autophagosome initiation directly on the cargo, distinguishing it from starvation-induced autophagy (Figure 1C). This CALCOCO2-dependent initiation of the ULK1 complex also occurs during mitophagy [50], with both xenophagy and mitophagy sharing a dependence on TBK1 [44,49–51]. Such integration of cargo detection and autophagy induction via an autophagy receptor has also been described for SQSTM1, which contains an RB1CC1-interaction region. Phosphorylation of this domain increases interaction with RB1CC1, resulting in recruitment of the ULK1 complex and autophagosome biogenesis on ubiquitin condensates [52]. During mitophagy, OPTN directly interacts with ATG9A to initiate the *de novo* synthesis of autophagosomal membranes [53]. It is therefore interesting to speculate that each autophagy receptor might induce *in situ* autophagosome biogenesis during xenophagy.

### Pathogen-specific xenophagy: lessons from mycobacteria

Much of our understanding on the mechanics of xenophagy derives from the analysis of *Salmonella*. Although *Salmonella* has adapted to replicate within vacuoles, its active invasion into epithelial cells results in a portion of bacteria that enter the cytosol and induce xenophagy. This lends *Salmonella* to be an excellent model organism for the study of xenophagy. Yet, as in any system, further unique aspects of xenophagy have been revealed through the study of diverse pathogens, as exemplified by the unique adaptations of *Mtb* to survive within cells.

Inducing autophagy in macrophages through starvation or IFNG/IFN-γ stimulation restricts the growth of *Mtb in vitro* [20]. Also, *ATG7* and *ATG14* are important for the restriction of *Mtb* growth in human macrophages [54]. Because whole body deletion of autophagy genes leads to perinatal or embryonic lethality, a common approach to support cell culture findings has been to generate cell type-specific knockouts, such as the myeloid-specific knockouts used in *Mtb* studies generated by breeding mice harboring a loxP flanked exon of *Atg5* with mice that express the Cre recombinase under the *Lyz2/LysM* promoter (*Lyz2-cre*) [55,56]. Mice lacking ATG5 in LYZ2+ cells are extremely susceptible to *Mtb* infection and succumb to *Mtb* infection by 40 days post-infection [55,57,58], similar to a mouse lacking IFN-γ signaling [59–61]. Yet, a subsequent study found that deletion of several other core autophagy genes including *Atg3*, *Atg7*, *Atg12*, *Atg14*, and *Atg16L1* using the same *LysMcre* myeloid-specific knockout strategy did not recapitulate the strong effect of *Atg5* deletion [57], and identified an autophagy-independent function of ATG5 in suppressing neutrophil-driven immunopathology during *Mtb* infection. In contrast, deletion of other essential autophagy genes, such as *Atg16L1* and *Atg7*, in LysM+ or ITGAX/CD11c+ cells results in increased susceptibility during chronic *Mtb* infection due to a role for autophagy in macrophages in controlling pro-inflammatory responses [62,63], which is a role for autophagy that is also observed at higher doses of *Mtb* infection [64]. These studies support an important role for autophagy in *Mtb* infection outcomes, and also reveal the importance of considering additional roles of autophagy proteins independent of autophagy. Mice that lack BECLIN1 or RB1CC1, but not ATG5, ATG16L1, and ATG7, in myeloid cells also display spontaneous immune activation and resistance to *Listeria* infection [65] whereas ATG16L1 promotes plasma membrane repair to limit *Listeria* spread independent of other autophagy proteins [66], indicating that individual autophagy proteins or complexes have functions in countering inflammation that cannot be explained by autophagosome-dependent pathways.

Although *Mtb* was once considered to reside in a sealed phagosome after uptake by macrophages, we now know that the virulence-associated type VII secretion system ESX-1 permeabilizes this membrane, enabling interaction with the cytosol [67–69]. ESX-1 is notably absent in attenuated strains such as *Mycobacterium bovis* Bacille Calmette-Guérin (BCG), and accordingly, ESX-1-deficient mycobacteria are not efficiently targeted by autophagy [55].

One consequence of this phagosomal permeabilization is the exposure of mycobacterial genomic DNA to cytosolic sensors. Early work demonstrated a role for the AIM2-like receptor IFI204 in invoking the cytosolic surveillance pathway in response to *Mtb* infection, followed by three subsequent studies that independently established cGAS (cyclic GMP-AMP synthase) as the primary sensor of this event [69–72]. Mycobacterial genomic DNA was found to be enriched in cGAS immunoprecipitates during infection, providing direct biochemical evidence for this pathway [70].

Although the precise mechanism by which DNA is released from the bacterium itself remains unresolved, key experiments have made a distinction between the permeabilization of the host phagosome and the translocation of DNA across the bacterial cell envelope. ESX-1-deficient *Mtb* does not normally recruit the autophagy machinery and elicit a type I interferon response; however, this mutant can be made to evoke these reactions if engineered to express the pore-forming toxin listeriolysin O (LLO), which specifically targets and permeabilizes the host phagosomal membrane [55,69]. This observation demonstrates that ESX-1-mediated membrane damage is the critical step that allows for the subsequent access of *Mtb* DNA to the cytosol. How this genetic material translocates across the mycobacterial cell envelope to then gain access to the cytosol remains an open question, with possible mechanisms including frank bacteriolysis, leakage from sub-lethally damaged bacilli, or intriguingly, the active release of DNA, consistent with the evolutionary benefit of evoking a host type I interferon response.

Detection of cytosolic mycobacterial DNA by cGAS leads to the production of cyclic dinucleotide GMP-AMP (cGAMP) and signaling through STING1 and TBK1. The subsequent action of ubiquitin ligases such as PRKN and SMURF1 promotes the ubiquitin-dependent targeting of a subset of bacilli for selective autophagy [34,55,70,73]. As with *Salmonella*, autophagy receptors including SQSTM1 and CALCOCO2 are recruited to mediate xenophagy [55]. Similarly, exposed glycans on damaged phagosomal membranes are detected by sensors such as LGALS8, which recruits the autophagy adaptor protein TAX1BP1 to the site of damage [28]. Therefore, in the case of *Mtb* infection, at least two signals, endomembrane damage and the cytosolic exposure of nucleic acids, act as triggers for xenophagy.

Intriguingly, *in vitro* infection studies demonstrate that only approximately one-third of *Mtb* bacilli that are internalized by macrophages are ultimately targeted to autophagosomes for degradation [34,55,70]. Considering the pronounced susceptibility and inflammatory phenotypes observed in autophagy-deficient mouse models, this discrepancy suggests that autophagy might play additional host-protective roles. One possibility is that mitophagy modulates the host response to infection by limiting the release of mitochondrial DNA and reactive oxygen species (ROS), which would otherwise trigger IFNA/IFNα and IFNB/IFNβ (type I IFNs; IFN-I) or inflammatory programmed cell death pathways that might be maladaptive during infection [74,75]. Supporting this, mice that lack the essential E3 ubiquitin ligase for mitophagy, PRKN, show greatly increased susceptibility to *Mtb* infection despite only a subset of bacilli becoming tagged with ubiquitin during *in vitro* macrophage infection [34].

## Evasion and subversion of autophagy

The relationship between autophagy and bacteria is complex, and depending on the pathogen and cellular context, it may result in either suppressing or promoting infection. In some cases, autophagy restricts pathogen replication by trapping and degrading the microorganisms and their replication niches through the xenophagy mechanisms described above. However, pathogens have acquired mechanisms to evade or exploit autophagy through co-evolution with the host [76,77].

### Bacterial virulence factors that interfere with xenophagy

Successful pathogens can block the initiation of autophagy by interacting with or modulating critical autophagy factors to avoid being targeted by xenophagy. For example, *Shigella* produces IcsB, which prevents its capture by the autophagy machinery by interfering with trafficking pathways [78], such as through fatty acylation of the ESCRT (Endosomal Sorting Complex Required for Transport) complex member CHMP5 (charged MVB protein 5) [79]. The causative agent of tularemia *Francisella tularensis* downregulates several autophagy genes such as BECN1, ATG5, ATG12, ATG16L2, ATG7, and ATG4A. In addition, *Franciscella* modulates the phosphatidylinositol-3-kinase/protein kinase B (PtdIns3K/Akt) pathway preventing autophagy; however, at later stages of cellular infection, the bacteria may benefit from being wrapped in autophagic vesicles [77,80]. *Salmonella* negatively modulates the AMPK-dependent activation pathway of mTOR, inhibiting autophagy initiation [81]. The *Shigella* effector OspB also activates MTORC1 inhibiting autophagy [82]. In contrast, the vacuolating toxin VacA of *Helicobacter pylori* inhibits MTORC1 and induces autophagy [83]. This finding may indicate that extracellular bacteria such as *H. pylori* are more tolerant of mTOR because xenophagy in response to alterations in host metabolism is less of a threat. The *Salmonella* type 3 secretion system (T3SS) effector SopF is critical for reducing LC3 localization to the PcV and promotes growth in vitro and extraintestinal dissemination in mice [84]. Other *Salmonella* effectors SseF and SseC interact with RAB1, interfering with the binding to its guanine nucleotide exchange factor (GEF), inhibiting RAB1 activation, and as a consequence, preventing the recruitment and activation of ULK1 [85]. Likewise, the *Shigella* effector VirA comprises a GTPase activating (GAP) domain, leading to RAB1 inactivation [86].

**Figure 2.**
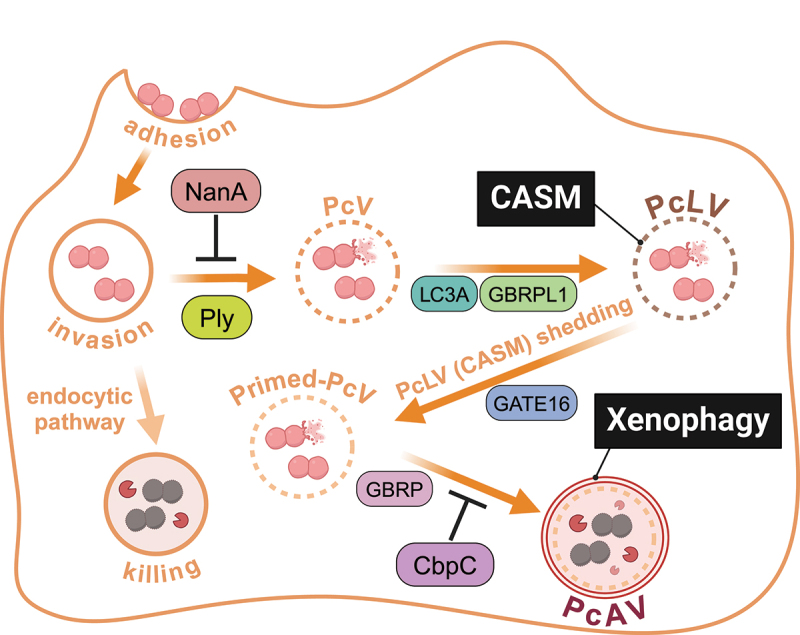
Invasion, CASM, and xenophagy during *Streptococcus pneumoniae* infection. Following the invasion of cells, *S. pneumoniae* exit the endocytic pathway due to the lytic action of pneumolysin (Ply). Initially, CASM is initiated, which depends on LC3A and GABARAPL1 (GBRPL1). The action of GABARAPL2/GATE16 (GBRPL2)) ensures these early pneumococci-containing LAPosome-like vesicles (PcLvs) become pneumococci-containing autophagic vesicles (PcAvs) following the induction of xenophagy. This process is inhibited by the bacterial protein CbpC. NanA is a pneumococcal sialidase that trims the sialylated glycans of endosomal or endo-lysosomal membrane proteins, suppressing Ply binding and promoting survival of the bacteria.

*Streptococcus pneumoniae* is a Gram-positive pathogen that colonizes the human nasopharynx and can cause life-threatening lower respiratory tract infections. The *S. pneumoniae* surface exposed protein CbpC acts as a decoy, inducing a form of selective autophagy that targets ATG14, via SQSTM1, for degradation. This loss of ATG14 suppresses autophagosome-lysosome fusion with CbpC, thereby promoting bacterial intracellular survival (Figure 2) [87]. *Listeria* produces several virulence factors that facilitate evasion. Internalin K cloaks the bacteria with protein complexes to avoid detection by autophagy [88]. The pore-forming toxin LLO induces autophagy [89], and in immunocompromised settings, generates an autophagy-dependent vacuole to survive in macrophages [90]. Although ActA, which mediates the generation of bacterial associated actin tails, is sufficient to prevent xenophagy in the cytoplasm by mobilizing the bacterium [91], other virulence factors such as the phospholipases are required for avoiding autophagy at later time points of infection [92]. Likewise, *Francisella tularensis* can lyse the autophagosomal membranes, escaping to the cytoplasm to continue replication. [93]

Given the selective pressure posed by xenophagy, it is not surprising that *Mtb* has been selected to use multiple strategies to evade this process. This includes *Mtb* proteins PE_PGRS20 [94] and PE_PGRS47 [95], which inhibit autophagy [96], and the virulence-associated lipid phthiocerol dimycocerosate, which confers resistance to autophagy and the autophagy-related pathway LAP discussed below [97]. Similarly, the aggregation of cytosolic mycobacteria to form cords may serve to shield them from selective autophagy [98]. Perplexingly, *Mtb* also produces factors such as Rv1468c that appear to promote targeting to selective autophagy [99]. The existence of these factors suggests that there may be a tradeoff between bacterial survival and immunopathology that is carefully balanced by *Mtb* to persist within its host and establish chronic, immunologically silent infection.

Interfering with ubiquitin and ATG8 proteins represents another mechanism of autophagy evasion. The polysaccharide capsule of *Burkholderia* blocks the association of RNF213 and its ubiquitin esterase, TssM, reverses RNF213-mediated LPS ubiquitination [100], whilst BopA prevents LAP [101]. In contrast, *Shigella* uses IpaH ubiquitin ligases to degrade RNF213 and LUBAC, effectively dampening ubiquitin-dependent eat me signals [13,102,103].

*Legionella pneumophila* is an intracellular Gram-negative bacterium that causes a type of pneumonia called Legionnaire’s disease. *Legionella* resides in an ER-derived vacuole that is heavily ubiquitinated [104,105], and therefore, should be a robust target for xenophagy. However, *Legionella* employs multiple strategies to prevent autophagic recognition of the vacuole in which it resides. The bacterial effector protein RavZ globally disrupts autophagy by irreversibly cleaving lipidated ATG8 proteins from autophagosomal membranes [106]. The SidE family of effectors prevents the binding of autophagy receptors to ubiquitin molecules associated with the vacuole [107–109]. Additionally, the *Legionella* effector LpSpl is a sphingosine-1 phosphate lyase that restrains autophagy, [110] and the effector Lpg1137 is a serine protease that cleaves STX17 to disrupt autophagy [111]. The multifaceted and biochemically distinct mechanisms by which *Legionella* inhibits autophagy likely reflect the importance of this pathway in cell-autonomous host defense in unicellular protozoa, which are the natural hosts for *Legionella* in their freshwater environments.

Of note, infections by a given bacterial pathogen do not always occur in isolation. Co-infections by viruses that divert the autophagy machinery, such as human immunodeficiency virus (HIV) and measles virus, increase the intracellular burden of *Mtb* and *Salmonella* [112,113]. It is possible that certain bacteria have adapted to the presence of other pathogens.

### Autophagosome-like replicative niches

Some pathogens hijack autophagy to form a specialized niche favoring their own replication. *Coxiella burnetii*, a Gram-negative intracellular bacterium that causes Q fever in humans and other animals, generates large acidic replicative vacuoles with degradative properties [114]. These vacuoles are strongly marked by LC3 and BECN1 [115–117] and by RAB7 and RAB24, two RAB GTPases involved in the fusion of autophagosomes with lysosomal compartments [116,118–122]. Autophagy activation, such as through amino acid deprivation, favors the replication of this bacterium, resulting in sustained infection [119].

*Coxiella* replication is not severely affected when essential autophagy genes are inactivated in the host cell; however, the vacuoles in which *Coxiella* replicates are smaller in size and vacuoles containing bacteria that were generated through independent uptake events do not undergo homotypic fusion [123,124]. Therefore, modulation of the autophagy machinery may be most important for microbial persistence or multicellular immune responses rather than short-term bacterial replication. The *Coxiella* effector protein Cig2 (CvpB) is essential for subversion of host autophagy [123]. *Coxiella cig2* mutant bacteria reside in vacuoles that do not accumulate LC3 and display a multi-vacuolar phenotype resulting from a defect in homotypic fusion of *Coxiella*-containing vacuoles [125]. The Cig2 protein disrupts the activity of the host phosphatidylinositol 5-kinase PIKfyve, which alters phosphatidylinositol 3-phosphate levels on the vacuole membrane [126]. Although *Coxiella* resides in a hydrolytic lysosomal-like compartment, the bacteria resist degradation. Although this bacterium remains in the large replicative niche during most of its infection cycle, the integrity of the PcV is altered [127]. Electron microscopy studies have demonstrated that the PcV membrane is pierced and disrupted with the resulting loss of protons and pH increase. Thus, these alterations likely contribute to bacterium survival.

The Gram-positive coccus *Staphylococcus aureus*, a skin commensal that can cause life-threatening invasive infections, also generates specialized LC3-labeled compartments. Transit through these autophagic-like vacuoles favors bacterial growth [128]. *S. aureus* infection prevents fusion of these compartments with lysosomes, inhibiting autophagosome maturation. Afterward, the bacterium escapes into the cytoplasm and induces a caspase-independent cell death, leading to bacteria spreading and infection of neighboring cells. Secretion of a bacterial hemolysin, the alpha-hemolysin, activates autophagy in Chinese hamster ovary cells [129]. Induction of autophagosomes has also been shown in infected bovine mammary epithelial cells, favoring intracellular replication [130]. This role of autophagy in favoring bacterial growth is supported by a study showing that inhibition of autophagy by overexpression of protein kinase C (PKC) inhibits *S. aureus* intracellular replication [131]. However, this bacterium uses several mechanisms to escape from autophagy degradation [132,133], either by preventing autophagosome maturation, as mentioned above, or by inhibiting the autophagic flux via IsaB, promoting transmission of methicillin-resistant *S. aureus* [134]. *S. aureus* inhibition of autophagosome maturation can be mediated in part by the phosphorylation of ATG5 by the mitogen-activated protein kinase 14 (MAPK14) [135].

In a manner similar to vacuoles containing internalized *Salmonella*, *Listeria*, and *Shigella*, LGALS8 is targeted to *S. aureus* damaged phagosomes. However, autophagy induced through this mechanism degrades innate immune proteins rather than *S. aureus* [136]. In *Listeria*-infected macrophages, LGALS3 protects the bacterium by suppressing the autophagic response [137]. In addition to modulating autophagy, galectins promote repair of damaged membranes by recruiting the ESCRT machinery [138,139]. These findings highlight how pathogens hijack the host machinery for membrane repair to maintain their replicative niches.

## Autophagy-bacteria interplay in plants

**Figure 3.**
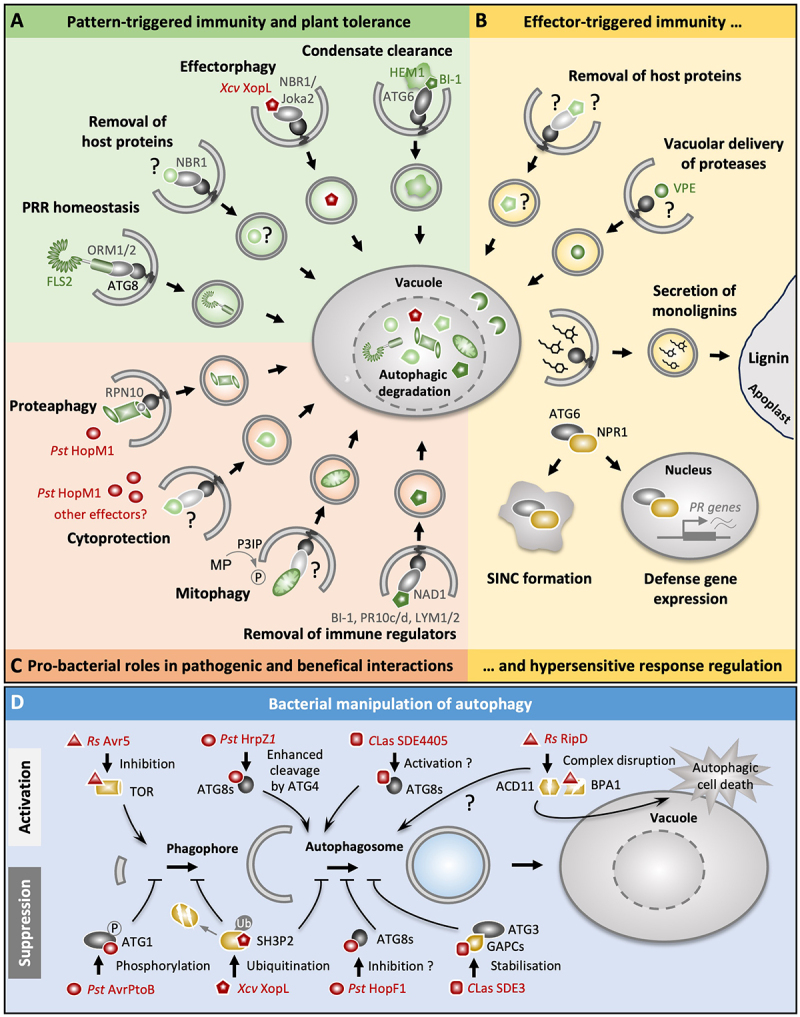
Autophagy mechanisms and functions in plant-bacteria interactions. (A) Autophagy is involved in pattern-triggered immunity and plant tolerance mechanisms. Selective autophagy mediates the turnover of the pattern recognition receptor FLS2 via the cargo receptors ORM1 and ORM2 and contributes to NBR1-mediated basal resistance responses that likely rely on the removal of as yet unknown host proteins. The cargo receptor NBR1/Joka2 is also able to directly target the bacterial effector protein XopL of *Xanthomonas campestris pv. vesicatoria* in a process referred to as “effectorphagy.” In addition, autophagy facilitates the removal of detrimental ER-associated HEM1 condensates via the interaction of ATG6 with the HEM1-binding partner BI-1, leading to improved tissue health during bacterial infection. (B) Autophagy contributes to effector-triggered immunity (ETI) and the regulation of the hypersensitive response (HR). Autophagy has been linked both to the promotion and restriction of the HR, yet the ATG8-interacting cargo receptors and degraded host proteins responsible for the associated cell death and survival activities remain largely unknown. However, non-catabolic functions of autophagy play important roles in ETI and HR processes. Autophagosomes mediate the vacuolar delivery of the death-promoting VPE protease, although the direct link to HR execution during avirulent bacterial infection awaits to be demonstrated. Autophagic vesicles are also involved in the secretion of monolignol precursors, which are required for the formation of a lignin barrier in the cell wall to restrict cell death and pathogen spread. Furthermore, the autophagy component ATG6 promotes the formation of NPR1-dependent condensates (SINCs) to limit immunogenic cell death to infected tissue. Interaction of ATG6 with NPR1 also increases NPR1 pools in the nucleus and subsequently, leads to enhanced defense gene expression and resistance against avirulent bacterial infection. (C) Autophagy has pro-bacterial functions both in pathogenic and symbiotic relationships. During *Pseudomonas syringae pv. tomato* (*Pst*) infection, the effector HopM1 inactivates proteasomes and triggers their autophagic degradation (proteaphagy), leading to the suppression of SA-dependent defense responses. HopM1 is also involved in the effector-mediated activation of autophagy that could help maintain cellular survival during the biotrophic phase of the bacterial infection. However, the exact mechanisms, cargo receptors and/or autophagic substrates mediating such pro-bacterial cytoprotective functions, are not known. During beneficial rhizobial infection of the legume *Medicago truncatula*, the selective degradation of damaged mitochondria seems to provide carbon skeletons and nitrogen for bacteroid development and formation of nitrogen-fixing cells. These processes are triggered by myotubularin phosphatase (MP)-mediated dephosphorylation of PtdIns3P on the developing autophagosome. Successful rhizobial colonization of *Medicago truncatula* involves also the MtNAD1-mediated removal of immunity-related proteins. (D) Bacterial pathogens use multiple effector-based strategies to subvert and hijack autophagy processes. Autophagy activation is mediated by the *Ralstonia solanacearum* (*Rs*) effector Avr5 through TOR inhibition or by the *Pst* HrpZ1 and *Candidatus* Liberibacter asiaticus (*C*Las) SDE4405 effectors via binding to ATG8 proteins. The *Rs* effector RipD activates autophagy by unknown mechanisms and influences the homeostasis of the ACD11-BPA1 complex by competitive binding to BPA1. While low levels of RipD promote ACD11 accumulation and cell death suppression, high levels lead to ACD11 and BPA1 degradation and the induction of autophagy-dependent cell death. Autophagy suppression is achieved by *Pst* AvrPtoB through ATG1 phosphorylation, by *Xcv* XopL through ubiquitination and subsequent proteasomal degradation of the autophagy regulator SH3P2, and by *Pst* HopF1 through ´inhibitory´ interaction with a subset of ATG8 proteins. Finally, *C*Las SDE4405 interacts with GAPCs, which prevents their degradation by ATG8 and likely promotes their inhibitory effect on the autophagy protein ATG3. See text for further details. Graphical elements are partly adapted from Kushwaha et al. [147].

As animals, plants are colonized by a large diversity of bacteria that form pathogenic, symbiotic, or commensal relationships with their host. While some bacterial pathogens cause diseases with severe consequences for plant fitness and productivity, beneficial bacteria can promote growth and stress tolerance to abiotic and biotic stimuli. In addition, there is emerging evidence that commensal bacterial communities are crucial for plant health and survival in various environmental settings [140,141]. Hence, plant-bacteria interactions strongly impact crop production and food security and contribute to ecosystem functioning and sustainability [142]. Due to this relevance, understanding the molecular mechanisms of how plants discriminate between and shape their responses to pathogenic and non-pathogenic/beneficial bacteria and how bacteria manipulate the host to their benefit has been in the spotlight of plant research for decades [143–146]. In this context, autophagy is increasingly recognized as a central pathway with multifaceted roles during plant-bacteria interplay. This section highlights recent advances in dissecting the anti- and pro-bacterial roles of autophagy as well as deciphering the bacterial strategies to subvert or hijack autophagy processes for plant colonization and infection (Figure 3).

### The plant immune system

Plants recognize and respond to bacterial challenge with the help of an innate immune system that comprises two interdependent and mutually potentiating branches of pattern- and effector-triggered immunity (PTI/ETI) for recent reviews, see [148,149]. PTI builds on PRRs at the cell surface to detect pathogen/microbe-associated molecular pattern (PAMP/MAMP, e.g., bacterial flagellin and elongation factor Tu) or host-derived damage-associated molecular patterns (DAMPs) and other immunogenic molecules. PRR activation initiates downstream signaling cascades resulting in transcriptional reprogramming and the production of antimicrobials to restrict bacterial proliferation. In contrast to their animal-infecting counterparts, plant-associated bacterial pathogens invade the extracellular space, yet secrete a large repertoire of effector proteins into the plant cell to suppress PTI and modulate cellular functions for enhanced virulence. In turn, the NLR class of intracellular immune receptors detect effector proteins or their activities on host proteins resulting in the induction of a more rapid and robust immune response than PTI. Such NLR-governed ETI is typically associated with a localized programmed cell death (PCD) reaction, known as the hypersensitive response (HR), which, however, can be dispensable for effector-triggered antibacterial resistance [150]. PTI and ETI activation also leads to systemic acquired resistance (SAR) responses to protect healthy tissue outside of the primary infection sites and in distal leaves from secondary microbial infections [151].

### Autophagy in ETI and HR regulation

Autophagy has been linked to various aspects of ETI during bacterial infection (Figure 3B). Initially, autophagy was found to play a dual role in the regulation of the HR upon challenge with avirulent strains of *Pseudomonas syringae* pv. *tomato* (*Pst*) DC3000. Infection assays in *Arabidopsis* knockout mutants of several core *ATG* genes resulted in reduced and delayed immunogenic cell death during the time course of the HR conditioned by different NLR proteins (e.g., RPS4 and RPM1) [152–155]. Such positive role of autophagy in HR promotion was further supported by the analysis of *Arabidopsis* plants with enhanced autophagy levels due to loss of cytoplasmic glyceraldehyde-3-phosphate dehydrogenases (GAPCs) or overexpression of a constitutive active form of the small GTPase RabG3b [154]. In contrast, accelerated spreading of cell death into healthy tissue was observed several days after avirulent *Pst* infection in *atg* mutants, indicating the contribution of autophagy to the confinement of HR lesions [156,157]. The cytoprotective activities of autophagy were previously linked to the removal of harmful components, the negative regulation of salicylic acid (SA) signaling, and/or the attenuation of ER stress caused by activation of NPR1 (nonexpressor of pathogenesis-related proteins 1)-dependent systemic immune responses [152,157,158]. In this context, ATG6 (BECN1) was recently found to interact with NPR1 and to promote the formation of SA-induced NPR1 condensates (SINCs) [159], which contribute to cell survival by sequestering ETI (e.g., NLRs) and stress-related components [160]. In addition, the observed co-localization of ATG8 and the cargo receptor NBR1 with SINCs implies the possibility that autophagy is involved in the turnover of condensates or their content [160]. Notably, the apparent stabilizing effect of ATG6 binding on NPR1 protein levels was also found to enhance nuclear NPR1 pools, which resulted in increased *pathogenesis-related* (PR) gene expression and improved resistance to the avirulent bacterial strain *Pst* DC3000 (AvrRps4) [159]. Because ATG6-promoted SINC formation dampened the HR, the observed growth restriction seems to be largely uncoupled from autophagy-dependent immunogenic cell death [159].

However, the exact mechanisms and spatiotemporal dynamics underlying the complex interplay of autophagy with ETI-associated HR and resistance responses are far from being understood. In particular, it remains to be investigated if and how distinct selective autophagy pathways contribute to infection-induced cell fate decisions, e.g., through context-dependent elimination of negative and positive determinants of HR. Intriguingly, recent reports point to an important role of autophagy as a membrane trafficking pathway in HR promotion and restriction. Autophagic vesicles were found to mediate the secretory transport of monolignols to the apoplast, where they are required for lignin deposition and subsequent physical limitation of cell death and pathogen spread [161]. In addition, autophagosome-mediated trafficking was shown to be necessary for the vacuolar delivery of VPE (vacuolar processing enzyme) during starvation-induced PCD in potato [162]. VPE is a well-characterized protease with caspase-like activities required for developmental and pathogen-induced cell death including the HR during virus infection [163,164]. However, it needs to be validated if the autophagic trafficking of VPE is causal for the (partial) autophagy-dependency of immunogenic cell death during avirulent *Pst* DC3000 infection in *Arabidopsis*.

### Autophagy in PTI and basal resistance

While autophagy has well-established roles in basal resistance against various pathogen classes including viruses and necrotrophic fungi [147,165], the contribution of autophagy processes to immune responses during virulent bacterial infection remained unclear. The initial analysis of several *atg* mutants revealed markedly enhanced resistance against hemi-biotrophic *Pst* DC3000 which was linked to elevated SA levels and enhanced SA-dependent defenses in response to autophagy-deficient conditions [166]. Notably, PTI responses like mitogen-activated protein kinase (MAPK) signaling and callose deposition were unaltered in the *atg* mutants [166], suggesting no direct involvement of autophagy in the PRR signaling pathway. However, these observations seem to contrast the proposed role of autophagy in the homeostasis of the FLS2 (flagellin-sensing 2) PRR [167] (Figure 3A). FLS2 was shown to be targeted for autophagic degradation by the selective cargo receptors orosomucoid 1 (ORM1) and ORM2, resulting in FLS2 hyperaccumulation and enhanced bacterial resistance in ORM1/2 RNAi *Arabidopsis* plants as well as diminished FLS2 levels and increased bacterial growth in ORM overexpressing *Arabidopsis* lines [167]. Furthermore, the PRR co-receptor and key immune regulator BAK1 (brassinosteroid-associated kinase 1) have been implicated in the negative regulation of autophagy through phosphorylation of the core autophagy protein ATG18a leading to compromised autophagosome formation [168]. However, this regulatory process has thus far only been described during infection with the necrotrophic fungus *Botrytis cinerea* and may be exploited by the pathogen to impair autophagy-mediated defenses for cell death and disease promotion. Hence, it remains to be investigated whether BAK1-mediated suppression of autophagy is involved in the regulation of FLS2 levels and/or resistance responses upon bacterial recognition. In case of the necrotrophic bacterium *Dickeya dadantii*, autophagy was assigned a rather indirect role in immune responses through modulation of hormone defense signaling [169]. While autophagy stimulation in *Atg8a* overexpressing *Arabidopsis* lines resulted in slightly reduced disease symptoms, *atg2* knockout plants showed enhanced susceptibility, most likely due to increased SA signaling that repressed the jasmonic acid (JA) defense pathway known to contribute to *Dickeya dadantii* growth restriction [170,171].

Mounting evidence has accumulated that NBR1-mediated selective autophagy is an integral part of anti-bacterial responses (Figure 3A). *nbr1* knockout mutants showed enhanced *Pst* DC3000 growth and displayed more pronounced disease progression and water-soaked lesions compared to *Arabidopsis* wild-type plants. Consistent with this, transient expression of *NBR1* in *Nicotiana benthamiana* leaves counteracted waterlogging in the apoplastic space [172], which is induced by the *Pst* effector HopM1 to enhance bacterial proliferation [173]. Since HopM1 levels remained largely unaffected by *NBR1* expression, it was speculated that NBR1 is involved in the removal of as yet unknown negative immune regulators or specific host components recruited by HopM1 for water-soaking [172]. Intriguingly, NBR1 was recently shown to directly target the effector protein XopL of *Xanthomonas campestris pv. vesicatoria* (*Xcv*) in a process termed “effectorphagy” [174], which resembled the NBR1-mediated degradation of viral proteins and particles as part of an antiviral plant defense pathway [175,176]. Hence, NBR1 has evolved as a conserved selective cargo receptor for xenophagy processes, targeting bacterial and viral components and/or the entire pathogen in different eukaryotic organisms [177].

Besides its role in resistance mechanisms to limit bacterial growth, autophagy has been implicated in plant tolerance against *P. syringae* and *X. campestris*. In general, tolerance is a host defense strategy that aims at minimizing tissue damage and disease severity irrespective of pathogen proliferation. A recent paper showed that hematopoietic protein 1 (HEM1) and its binding partner Bax-inhibitor 1 (BI-1) mediate the formation of harmful ER-associated condensates upon SA-accumulation during virulent bacterial infection [178]. While these condensates sequester lipid-metabolic enzymes and disturb lipid homeostasis, they are proposed to be removed by autophagy through the known interaction of BI-1 with ATG6, thereby alleviating the negative impact on tissue health[178] (Figure 3A). Notably, HEM1 condensates are also formed during ETI, but in this case were linked to the dampening of the HR through the restriction of pro-death immune gene translation [179]. Whether the ETI-associated HEM1 condensates are also subject to autophagic degradation or even promoted by ATG6 as shown for SINCs awaits further analysis.

### Pro-bacterial functions of autophagy

In agreement with the increased resistance response of autophagy-deficient mutants, autophagy stimulation by chemical inhibition of the TOR kinase or genetic overexpression of *ATG5* supported *Pst* DC3000 proliferation in *Arabidopsis* [172]. This beneficial effect was linked to the selective autophagic degradation of proteasomes in response to their inhibition by bacterial effectors that directly bind to proteasomal subunits. The *Pst* DC3000-induced proteaphagy pathway was found to be critical for the efficient block of proteasome function [172], resulting in the suppression of PTI and SA-dependent defense responses [180]. Thus, the observed elevation of proteasome activity in *atg5* mutants [172] might further add to the causal explanation of the enhanced resistance under autophagy-deficient conditions. Notably, the *Xcv* effector XopJ was also shown to interfere with the proteasome [181], yet it remains to be determined whether the suppression of proteasome activity engages proteaphagy during *Xanthomonas* infection. Similarly, the consequences of pathogen-triggered proteaphagy and impaired proteasome activity for host cell health and/or cell death processes during infection require further investigation. In this context, the observed effector-induced enhancement of ´bulk´ autophagy could serve hemibiotrophic bacteria by maintaining cellular survival during the biotrophic phase for the establishment of infection before switching to necrotrophy. Such cytoprotective function of autophagy is well established during virus infection to increase plant health and life span for particle production and potential transmission [175,176,182]. Alternatively, autophagy might contribute as death-promoting and/or nutrient recycling mechanism to processes associated with the transition to the necrotrophic phase, e.g., during infection with *Ralstonia solanacearum* [183,184].

### Autophagy in beneficial bacterial interactions

There is an increasing recognition of autophagy processes contributing to the regulation of plant relationships with beneficial bacteria [185] (Figure 3C). Legumes form a symbiosis with nitrogen-fixing soil bacteria, known as rhizobia, resulting in the formation of root nodules in which atmospheric N_2_ is converted to plant-available ammonium in exchange for host photosynthates. Establishment of the symbiotic partnership and nodule organogenesis relies on complex developmental programs and molecular crosstalk. In particular, the formation and maintenance of specialized infected cells in the nodules, where the endocytosed rhizobia colonize and differentiate into nitrogen-fixing bacteroids, need to be tightly controlled to prevent targeting of the symbiont by the host immune system. The role of autophagy in legume-rhizobium interactions is still poorly understood, but core regulators like TOR, PtdIns3K and ATG6 have been connected to early nodulation events [186,187]. While autophagy-independent functions of TOR and PtdIns3K complexes might also contribute to the observed effects in the respective *Phaseolus vulgaris* RNAi lines, the recent analysis of myotubularin phosphatase (MP) mutants in *Medicago truncatula* uncovered mitophagy as a critical process for symbiotic nitrogen fixation [188]. MP was found to dephosphorylate PtdIns3P on autophagosomes and to participate in the recycling of damaged mitochondria in the infection zone. It was speculated that mitophagy-derived carbon skeletons and nitrogen support bacteroid development and the transformation of infected into nitrogen-fixing cells [188]. Intriguingly, autophagy mechanisms have recently been directly linked to the suppression of immunity in nodules of *Medicago truncatula* [189]. Knockout mutants of *MtNAD1* (nodules with activated defense1) displayed defective nodules with hyperactivated immune responses, a phenotype also observed in autophagy-deficient mutants lacking MtATG7. MtNAD1 was found to interact with MtATG8 and to mediate the selective degradation of immunity-related proteins (e.g., MtBI-1a, MtPR10c/d, MtLYM1/2) during rhizobial colonization of developing nodules.

In addition to the emerging importance of autophagy for symbiotic bacterial interactions, there are first indications that autophagy may also contribute to the formation of the root microbiome. Multi-omics analysis in autophagy deficient *Arabidopsis* mutants indicated that autophagy regulates the assembly and functional diversity of bacterial communities in the rhizosphere and endosphere, which was linked to the impact of autophagy on cell wall, defense, and root exudates[190]. Future studies will reveal if and to what extent the autophagy-mediated modulation of beneficial bacteria in the root microbiome has direct consequences for plant growth and fitness.

### Bacterial manipulation of autophagy

The multifaceted anti- and pro-microbial functions of autophagy during plant-bacteria interactions imply the evolvement of several bacterial factors and strategies to manipulate autophagy processes for the benefit of host colonization and proliferation. Indeed, the systematic screening of *Pst* effector proteins for direct interaction with core ATG proteins suggested different mechanisms to modulate autophagy [191] (Figure 3D). For instance, the effector protein HrpZ1 enhanced autophagic activity by promoting ATG4-mediated cleavage of various Arabidopsis ATG8s, whereas autophagy was suppressed by binding of HopF1 to another ATG8 subset or the phosphorylation of ATG1 by AvrPtoB. Strikingly, both the stimulation or inhibition of autophagy had a positive effect on bacterial virulence [191]. In line with these findings, the *Pst* effector HopM1 was shown to enhance autophagy flux and induce the selective autophagic degradation of proteasomes for enhanced pathogenicity. In contrast, the *Xcv* effector XopL triggers the proteasomal degradation of the positive autophagy regulator SH3P2, resulting in autophagy suppression to prevent NBR1-mediated antibacterial immune responses [174]. Recent reports further proposed the manipulation of autophagy by effectors of the phloem-limited bacterium “*Candidatus* Liberibacter asiaticus” (*C*las) associated with the destructive citrus Huanglongbing (HLB) disease. The effector SDE3 was demonstrated to inhibit autophagy through interaction with the negative autophagy regulators cytosolic glyceraldehyde-3-phosphate dehydrogenases in *Citrus sinensis* (CsGAPCs), which prevented their degradation by ATG8 proteins and likely favored their inhibitory effect on ATG3 proteins [192]. *C*Las effector SDE4405, however, interacted with ATG8 proteins resulting in the promotion of autophagy [193]. Transgenic expression of both effectors favored bacterial infection with *CLas* and *Xanthomonas citri subsp. citri* (*Xvv*) in citrus [192,193], yet whether these pro-bacterial effects are indeed causally connected to the opposing impact on autophagy activity or at least partly mediated by an autophagy-independent suppression of antibacterial defenses requires further clarification. Finally, effector proteins from *Ralstonia solanacearum* also modulate autophagic processes. While the Awr5 effector was identified as an autophagy activator acting through TOR inhibition [183], the underlying mechanisms of the autophagy-inducing activity of the RipD effector remained unclear. Notably, RipD interrupted the homeostasis of the BPA1-ACD11 complex in a dose-dependent manner through competitive binding to BPA1, which seems to influence autophagy-related cell death control [184]. Low levels of RipD favored ACD11 accumulation and suppressed cell death, whereas high levels of RipD resulted in ACD11 and BPA1 degradation and subsequent induction of autophagy-dependent PCD.

Together, these examples of both effector-mediated autophagy enhancement and inhibition strongly illustrate the relevance of autophagy processes for host resistance and bacterial pathogenesis. Hence, effectors are likely secreted in a highly coordinated fashion during the bacterial life cycle to precisely alter autophagy levels for infection and proliferation. To what extent factors of beneficial bacteria are capable of directly modulating host autophagy for establishing their commensal or mutualistic relationship with plants is an exciting area of future research.

## Intersection between immune recognition of bacteria and autophagy

**Figure 4.**
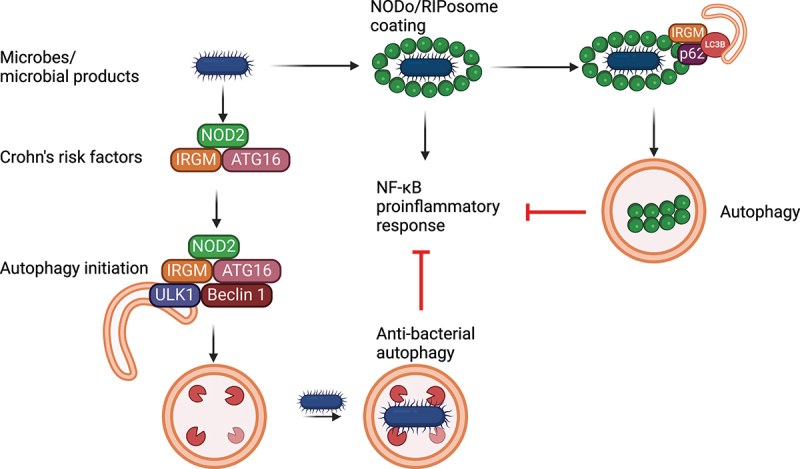
Crohn disease risk genes in anti-bacterial autophagy and innate immune homeostasis. NOD2, ATG16L1, and IRGM, key CD-associated proteins, interact with autophagy machinery to initiate antimicrobial autophagy (xenophagy). Simultaneously, bacterial infection induces NOD and RIPK2 oligomerization, forming NODo/RIPosomes that drive NFKB/NF-κB-mediated inflammation, a critical antibacterial response. The system is tightly regulated by selective autophagy, which degrades NODo/RIPosomes (inflammophagy) to prevent excessive inflammation and maintain innate immune homeostasis.

Identification of genetic loci conferring altered susceptibility to various human diseases has provided valuable opportunities for improved insight into mechanisms regulating both health and disease. In particular, Crohn disease-associated genetic variants in autophagy pathways sparked the active and productive investigation for roles of autophagy in the intestine [194–200]. Crohn disease and ulcerative colitis are the two major disease subtypes comprising inflammatory bowel disease (IBD), a disease associated with recurring intestinal inflammation generally thought to be secondary to a dysregulated response to intestinal microbiota. The continuous exposure to a high density of resident microbes in the intestinal lumen poses substantial challenges with respect to gut and systemic homeostasis. Mechanisms regulating microbial clearance in a manner that minimizes inflammation and tissue injury are therefore particularly important in intestinal tissues. Autophagy is one such potential mechanism, as it increases the clearance of intracellular microbes while reducing inflammasome activation, and in turn, various inflammatory pathways triggered by their products (e.g., IL-1B). IBD-associated variants of genes in the autophagy pathway generally lead to reduced autophagy [201–204] (Figure 4). This can be mediated by a loss of function in genes promoting autophagy, such as *ATG16L1* and the IFN-inducible GTPase gene *IRGM*, or a gain in function in genes inhibiting autophagy, such as *MTMR3* [194,195]. Additional IBD-associated genes suppressing autophagy include *LRRK2* [205,206]. The genes inhibiting autophagy have been less investigated but may ultimately provide a beneficial means by which to modulate autophagy levels therapeutically. In addition to the IBD-associated variants that directly regulate autophagy, there are a number of IBD-associated genetic variants that modulate genes encoding proteins that regulate sensing of bacteria through PRR-initiated pathways, which in turn, can regulate autophagy. An example is variants in the gene encoding NOD2 (nucleotide binding oligomerization domain containing 2). IBD-associated genetic variants in PRRs lead to both elevated and reduced PRR-initiated outcomes, such that either an excessive or inadequate innate response can lead to an inappropriate response to intestinal microbes [207,208]. This highlights the importance of regulating the threshold of these pathways in the intestine. In the case of IBD-associated genetic variants that lead to a loss-of-function of PRR responses, the attenuated induction of autophagy upon PRR stimulation reduces the ability to clear microbes within the intestine. The reduced ability to clear microbes can then lead to an increased microbial burden in intestinal tissues and to increased intestinal injury. While myeloid cells are key immune cells in clearing microbes and in promoting adaptive immune responses upon microbial challenges, and appropriate levels of autophagy are required for these functions, the functions of other cell types can also be regulated by IBD-associated genes through either intrinsic or extrinsic mechanisms. As such, autophagy regulates many different cell types within intestinal tissues in distinct manners that can impact IBD. In this section, we will discuss how autophagy and associated pathways integrate sensing of bacteria with regulation of bacterial numbers and inflammation, and how all of this can impact human diseases.

### Autophagy in Crohn disease and regulation of inflammation

Xenophagy targets multiple bacterial pathogens to the lysosome, including Crohn disease associated *Escherichia coli* [209]. As discussed earlier, bacteria that escape phagosomes or reside in ruptured phagosomes are poly-ubiquitinated by multiple host E3 ligases, such as LRSAM1, PRKN, SMURF, LUBAC, RNF166, and RNF213 [13,33–210] and then targeted for autophagy by autophagy receptor proteins such as SQSTM1 [211], CALCOCO2 [44,212], OPTN [45], and TAX1BP1 [213] that have ubiquitin and LC3-binding domains. One way that receptor-mediated xenophagy suppresses inflammation is by reducing bacterial burdens. Although intracellular bacterial pathogens have been selected through co-adaptation with their hosts to use mechanisms of blocking xenophagy [214], autophagy also maintains innate immune homeostasis independent of xenophagy by suppressing inflammation [63], in part by degrading proteins involved in inflammatory signaling pathways, such as inflammasome [215–218]. These multiple roles for autophagy likely account for why mutations in autophagy genes confer susceptibility to inflammatory diseases, including Crohns disease.

The first autophagy gene to be associated with Crohn disease was *ATG16L1* [196,197,219]. An ATG16L1 variant, T300A, that is prevalent in the human population confers a defective innate immune response to enteric bacteria resulting in increased numbers of intracellular bacteria [201,204,220,221], elevated secretion of the proinflammatory cytokines [220–222], and impaired antigen uptake and processing [201,223]. Heterozygosity is associated with increased NLRP3 inflammasome activity in mice and human myeloid cells [224,225]. Consequently, mice heterozygous for the T300A allele equivalent, but not homozygotes that are defective in LAP, display protection from *Salmonella* and *Listeria* infection, suggesting the variant has undergone balancing selection [225]. Myeloid cells in the intestinal environment are particularly effective at clearing bacterial challenges [226] and have higher levels of autophagy relative to myeloid cells in peripheral tissues [227]. Importantly, the levels of autophagy in intestinal myeloid cells depend upon the ongoing exposure to resident luminal bacteria; antibiotic treatment reduces autophagy levels and conversely, a slight increase in epithelial permeability and exposure to resident intestinal luminal bacteria increases autophagy levels in intestinal myeloid cells [227]. Autophagy levels in intestinal myeloid cells depend on bacterial recognition molecules such as NOD2 [227]; NOD2 can similarly regulate autophagy in peripheral myeloid cells [201,204,228]. In addition to immune cells, ATG16L1-mediated autophagy contributes to distinct functions within various epithelial cell lineages that impact protection and susceptibility to Crohn disease. The ability to induce optimal levels of autophagy in epithelial cells in response to various bacterial challenges depends on intact bacterial-recognition pathways [229]. The critical role of autophagy and ATG16L1 in secretory epithelial cells in preventing intestinal inflammation will be discussed below in the context of controlling extracellular pathogens. As such, Crohn disease-associated genetic variants in autophagy pathways have highlighted key roles for autophagy in distinct cells subsets that cooperate to regulate intestinal immune homeostasis, a tissue where there is ongoing exposure to a high density of resident microbes, as well as to the enhanced response needed upon exposure to enteric pathogens.

### PRR sensing of bacteria in Crohn disease and regulation of inflammation

Loss-of-function in PRR-initiated pathways has been observed with IBD-associated genetic variants in *NOD2*, *IL18RAP*, *LACC1*, *RNF186*, *ICOSLG/ICOSL*, and *INAVA* [199,200,230–235], thereby extending the disease-associated genetic variants which can ultimately lead to reduced autophagy. In particular, genetic variation in *NOD2* accounts for 20% of Crohn disease genetic risk, with three common variants [236]. Upon activation by MDP, NOD2 engages with RIPK2, leading to its ubiquitination and oligomerization into structures called RIPosomes [202,237,238], which are recruited to the bacteria [202], crucial for downstream inflammatory signaling, and are necessary for activation of NF-κB responses [202,237]. NOD2-RIPK2 activation not only induces inflammatory responses but also enhances antibacterial autophagy (xenophagy) and anti-inflammatory autophagy (inflammophagy) [201–204,239,240]. The building blocks for this pathway may have evolved early in metazoans because peptidoglycan recognition by a PRR in *Drosophila* mediates xenophagy against *Listeria* [241]. Mechanistically, upon activation, NOD2 interacts with IRGM and induces its K63-linked ubiquitination, which was found to be crucial for the recruitment and activation of core autophagy machinery including ULK1, ATG16L1, and BECN1 for anti-bacterial and anti-inflammatory autophagy [203] (Figure 4). In the presence of bacteria or MDP in epithelial cells, NOD2 directly engages with ATG16L1 on the plasma membrane to engulf bacteria [204]. In dendritic cells, NOD2- and ATG16L1-mediated autophagy is important for both xenophagy [228], antigen presentation, and the generation of major histocompatibility complex (MHC) class II antigen-specific CD4+ T cell responses [201,228].

There are 22 known NLR family members in humans and 34 in mice [242,243]. NLRP3, one of the most well-studied NLRs, senses a myriad of stimuli (bacteria, viruses, fungi, and parasites or their virulence activities) and promotes the assembly and activation of the inflammasome for defense against pathogenic microbes [242,243]. Unrestricted NLRP3 inflammasome activation can be detrimental and lead to multiple immune-mediated diseases and cancer [242,243]. Autophagy is triggered by the same stimuli that activate inflammasome and dampens the inflammasome to maintain immune homeostasis, representing a negative feedback loop [244,245]. Autophagy can indirectly regulate inflammatory products, such as preventing mitochondrial ROS and DNA accumulation through mitophagy [246–249], or directly sequester NLRP3 inflammasome and signaling components, such as through recognizing and degrading SQSTM1 and NLRP3 itself [218,250,251].

In addition to NLRP3, other NLRs regulate autophagy and antibacterial defense. NLRP6 was found to be important for autophagosome formation in intestinal epithelial cells, which was crucial for the secretion of mucus for anti-microbial defense [252]. NLRP4 colocalizes with GAS-containing autophagosome-like vacuoles where it directs Rho-actin signaling to promote autophagosome formation and maturation resulting in the killing of bacteria [253]. NLRX1 was shown to colocalize with mitochondria and was suggested to be a receptor for mitophagy [254]. Interestingly, *Listeria* hijacks and enhances this NLRX1-dependent mitophagy to decrease mitochondrial ROS production and thus promotes its own survival [254]. Taken together, multiple NLR proteins regulate autophagy for the modulation of bacterial infection. However, the roles for the majority of NLRs in immune responses are not well defined.

Disease-associated genetic variants leading to loss-of-function in PRR pathways have also provided insight into a variety of mechanisms leading to a deficiency in autophagy. For example, activation of NOD2 induces MTF1 (metal regulatory transcription factor 1), which in turn, regulates a number of metallothionein proteins, intracellular zinc, and autophagy [227]. Consistent with the ongoing stimulation of NOD2 in innate immune cells in intestinal tissues, metallothionein and intracellular zinc levels are higher in intestinal myeloid cells relative to bone marrow-derived myeloid cells and these levels increase in a NOD2-dependent manner with intestinal injury [227]. The zinc-dependent autophagy in macrophages highlights a potential mechanism by which oral zinc improves select measures in IBD patients [255] and benefits in patients with acute diarrhea in the developing world [256].

#### Regulation of autophagy by MTMR3 and Crohn disease

The IBD-associated gene *MTMR3* is a member of the myotubularin family. Active myotubularin members possess phosphoinositide 3-protein tyrosine phosphatase activity [257]. MTMR3 can inhibit autophagy by dephosphorylating PtdIns3P and PtdIns(3,4)P_2_ [257,274]. PtdIns3P is a key lipid mediator of membrane trafficking and signaling and participates in effector recruitment to autophagic membranes. MTMR3 was initially shown to inhibit constitutive autophagy in cells lines [258–260] and subsequently shown to inhibit the induced levels of autophagy observed with stimulation of PRRs in primary human macrophages [261]. The induced autophagy observed with both acute and chronic PRR stimulation, as occurs continuously in the intestinal environment, can in turn promote more effective intracellular microbial clearance. During early periods of PRR-induced autophagy, MTMR3 transiently re-localizes from the cytoplasm to the nucleus in human macrophages, thereby moving the inhibitory protein spatially away from the site of autophagy induction [261]. The ability of MTMR3 to inhibit PRR-induced autophagy in human macrophages requires both a catalytic-mediating cysteine within its phosphatase domain and an intact N-terminal PH-GRAM domain which can mediate binding to PtdIns3*P* [227]. Targeting these domains is therefore a means by which to reduce the activity of this autophagy inhibitor. The variant in *MTMR3* associated with increased Crohn disease risk results in increased MTMR3 expression, and in turn, reduced levels of autophagy in human macrophages [261]. As expected, this is associated with increased inflammasome activation and associated IL1B secretion, which further increases a range of other inflammatory cytokines [261]. These findings have highlighted the modulation of autophagy through a physiological variant in an autophagy inhibitor as a potential means to therapeutically regulate autophagy levels.

### IFN-inducible GTPases in autophagy-related host defense and inflammation

Many autophagy-related antimicrobial and inflammatory activities are executed by IFN-inducible GTPases. IFNs trigger production of four families of IFN-inducible GTPases: Immunity Related GTPases (IRGs), Guanylate Binding Proteins (GBPs), Myxovirus-resistance (Mx) proteins, and very large IFN-inducible GTPases (VLIG/GVIN). Whereas GVIN proteins remain uncharacterized, IRGs, GBPs and Mx proteins are well established executioners of cell-autonomous immunity in vertebrates [262]. Mx proteins provide exclusively antiviral defense. IRGs and GBPs on the other hand are equipped with versatile defense modalities directed at viruses, bacteria, fungi, and protozoa [262]. In addition to these roles in host defense, IRGs and GBPs have also emerged as critical regulators of inflammation.

#### Immune related GTPases (IRGs)

IRG proteins are divided into two subfamilies based on whether they contain the canonical GKS or noncanonical GMS sequence in their guanosine triphosphate (GTP)-binding pocket [262]. This division based on protein sequence also bifurcates the IRG family functionally. The GKS proteins, also known as “effector” IRGs, bind to cytosolic bacteria and to PcVs, where they facilitate the membranolytic or xenophagic destruction of pathogens [263–270]. GKS deposition on PcVs is assisted by the GMS (or IRGM) proteins and is dependent on the recognition of multiple patterns including the ATG8ylation of vacuolar membranes [271].

The genomes of most domesticated and wild-derived mouse strains encode 10 to 20 IRG genes, most of which belong to the GKS subfamily [272]. In contrast to mice, humans contain only one GKS gene, *IRGC*, expression of which is mostly limited to testis, and one GMS gene, *IRGM*, that is expressed more widely as five differentially spliced mRNAs (*IRGMa* to *IRGMe*) [273,274]. Allelic variation in human *IRGM* is associated with altered susceptibility to mycobacterial infections [275,276] as well as a number of immune-mediated disorders including Crohn disease [277–279], metabolic dysfunction-associated steatotic liver disease [280], ankylosing spondylitis [281], and sepsis [282]. Disease-linked human *IRGM* alleles contain one or more genetic variations that lie outside of the *IRGM* protein coding sequences, including a large deletion in the *IRGM* promoter region, and are associated with diminished expression [278,282,283]. Mouse studies demonstrate that *Irgm* deficiencies result in more severe inflammation in colitis models, increased mortality during experimental endotoxemia, and elevated susceptibility to bacterial infections [284–293]. Deletion of a single *IRGM* ortholog, mouse *Irgm1*, is typically sufficient for these inflammatory disease phenotypes to develop. Both mouse IRGM1 and human IRGM have overlapping functions in controlling mitochondrial dynamics [273,288,294,295] and autophagic flux [295–299]. Collectively, these reports indicate that mouse IRGM1 acts as the main functional ortholog of human IRGM and that *Irgm1-*deficient mice provide an accessible experimental model to study IRGM-associated human diseases.

Early studies of IRGM proteins were among the first to establish autophagy as a host mechanism to eradicate intracellular pathogenic bacteria. Loss of either human IRGM or mouse IRGM1 can diminish xenophagic clearance of *Mtb* or pathogenic *E. coli* from infected host cells *in vitro* [222,273,296,300], although it has been questioned whether mouse IRGM1 colocalizes with phagosomes containing *Mycobacterium bovis* BCG in IFN-γ-treated cells [301]. Mechanistic follow-up studies were mostly focused on human IRGM, which enhances autophagy initiation through engagement with ULK1 [203,302,303]. In addition, as previously noted, IRGM forms a complex with NOD2 and ATG16L1 to facilitate the nucleation or elongation of autophagosomes to capture bacterial pathogens [203]. Human IRGM also stimulates fusion with lysosomes through the recruitment of the membrane fusion protein STX17 to autophagosomes [304]. The reduction in bacterial burden through IRGM-dependent xenophagy simultaneously reduces inflammation by removing microbial ligands. IRGM proteins regulate inflammation and restrict mycobacterial and other bacterial infections through other autophagy dependent and independent functions. This includes a role in autophagic removal of inflammatory modulators, such as the NLRP3 inflammasome [250], NOD proteins, RIPK2, and self-assembling RIPK2 oligomers (RIPosomes) (Figure 4) [202].

IRGM proteins also regulate host defense through the control of mitochondrial homeostasis and IFN-I signaling pathways. Loss of human *IRGM* or mouse *Irgm1* expression disrupts mitophagy and leads to the accumulation of dysfunctional mitochondria [273,288,294,305–307]. Dysfunctional mitochondria release DAMPs like oxidized mitochondrial RNA and mitochondrial DNA, which can engage PRRs (cGAS-RIGI-TLR3) leading to the production of proinflammatory cytokines. In fibroblasts and epithelial cells, deletion of either human *IRGM* or mouse *Irgm1* results in sterile inflammation and IFN-I production that is primarily driven by the DNA-sensing cGAS-STING1 and RNA-sensing RIGI-MAVS signaling pathways [295,305]. Human IRGM, along with SQSTM1, also mediates autophagic degradation of cGAS and RIG-I in basal conditions [295]. Thus, in IRGM-deficient cells, both soiling of the cytoplasm with mitochondrial DNA/dsRNA and the presence of heightened DNA/RNA sensors contribute to IFN-I production [295,305]. Sterile induction of IFN-I is also observed in *Irgm1*-deficient primary macrophages, potentially due to disruption of mitophagy resulting in the shunting of mitochondrial RNA into endosomal compartments that contain the endosomal single stranded RNA sensor TLR7 [305].

While mitochondrial dysfunction appears to be a central cause of the inflammation associated with disrupted *IRGM* expression, peroxisome numbers in *Irgm1*-deficient macrophages are substantially diminished [308]. Peroxisomes are organelles that play key roles in lipid metabolism and provide a compartment for oxidative reactions. Their biogenesis is driven by the PPARA (peroxisome proliferator activated receptor alpha) pathway [309]. Treatment of *Irgm1*-deficient macrophages with PPARA agonists restored peroxisome numbers and lowered IFN-I expression to levels close to wildtype macrophages[308]. These observations suggest that IRGM regulates peroxisome homeostasis to suppress TLR7 activation and IFN-I expression in macrophages. The precise functional relationship between peroxisomal and mitochondrial dynamics orchestrated by IRGM proteins and how its disruption leads to TLR7 activation awaits further exploration.

Consistent with the induction of IFN-I expression, *Irgm1*−/− mice develop a type I interferonopathy associated with the production of autoantibodies and extensive tissue damage that involves lymphocytic infiltration in diverse tissues and organ atrophy. Deletion of the universal IFN-I receptor IFNAR completely reverses the autoimmune pathology of *Irgm1*−/− mice, whereas deletion of cGAS or STING1 only provides partial restoration of the animals’ health [305]. The increased susceptibility of *Irgm1*−/− mice to several bacterial pathogens including *Mtb, Listeria*, and *Salmonella* is reversed upon deletion of *IFNAR* [290,291]. Elevated IFN-I production in *Irgm1*−/− mice was shown to inhibit CD4+ T cell expansion during *Mtb* infections, thereby providing a mechanistic framework for the impact of *Irgm1* loss on host defense [290]. These findings are consistent with previous reports demonstrating the detrimental impact of IFN-I on host immunity to *Mtb* and several other bacterial infections [310–319].

It is possible that IRGM proteins are part of an ETI strategy. A primary function of IRGM proteins could be to eliminate viral, bacterial, and protozoan pathogens through cell-autonomous defense programs such as xenophagy. Because IRGM proteins simultaneously minimize an inflammatory response, any microbial counter-immune strategy disrupting IRGM function would trigger a localized inflammatory response. This localized inflammatory response could be greatly beneficial to the host, as it would elicit the infiltration and activation of immune cells and promote sterilizing immunity when xenophagy is inhibited. *Irgm1*−/− mice are indeed highly resistant to some viral infections [320,321].

#### Guanylate binding proteins (GBPs)

GBPs constitute a second family of IFN-inducible GTPases with prominent roles in inflammation and antibacterial host defense involving autophagy. The GBP family consists of 11 members in mice and 7 members in humans. Both mouse and human GBPs consist of an N-terminal catalytically active large G domain followed by helical middle and effector domains. Human GBP1, GBP2, and GBP5 and their mouse orthologs bear CaaX boxes at their C-termini resulting in protein prenylation, which is essential for GBP binding to host and microbial membranes [322]. The high degree of sequence homology between mouse and human GBPs, as well as experimental observations, indicate that GBP functions are conserved between these species [322].

The best studied GBP is human GBP1, an LPS-binding protein [323,324]. In the cytosol of infected cells, thousands of GBP1 molecules can form coatomers on the surface of Gram-negative bacteria [323,325,326]. Coating of bacteria is dependent on GBP1 polymerization [323], a process that requires energy generated through GTP hydrolysis [327,328]. Whereas monomeric nucleotide-free GBP1 exists in a safety-pin-like conformation [329,330], GTP binding and hydrolysis results in the unfolding of GBP1 into an outstretched conformation [331]. In its outstretched form, GBP1 releases its C-terminal fatty acid farnesyl group and forms polymers. The farnesyl tails of many GBP1 molecules form the polymer’s “greasy” core, whereas the G domains constitute the polymer’s outer ring [327,328]. These GBP1 polymers first dock to the bacterial surface [323] and then depolymerize to “drill” through the bacterial outer sugar coat consisting predominantly of the O antigen portion of LPS [322,323]. During this process, outstretched GBP1 dimers are released from the polymer into the bacterial outer membrane, where they become anchored via their C-terminal farnesyl tails [322,323,325,326]. The resulting GBP1 coatomer acts as a surfactant that breaks down the barrier function provided by the extensive sugar coat of the bacterial outer membrane [323]. This detergent-like activity of GBP1 allows for the IFN-inducible APOC3 (apolipoprotein C-III) to directly lyse GBP1-coated bacteria [332]. Bacteriolysis results in the release of LPS molecules [332,333] that are further aggregated by GBP1 and its paralog GBP2 to facilitate noncanonical Caspase-4 inflammasome activation [323,334].

CASP4 (caspase 4) activation is only one of several host cell death pathways induced by GBP1 [322]. Forced GBP1 production in the absence of IFNG stimulation induces Golgi fragmentation and kills the cell. To limit potential self-damage in uninfected IFN-primed cells, GBP1 is phosphorylated by the IFN-inducible kinase PIM1. Phosphorylated GBP1 is sequestered by the host protein 14–3–3σ. However, disruption of IFN signaling depletes the short-lived pool of PIM1 protein and triggers proinflammatory cell death [335]. Therefore, PIM1 acts as a guard that detects pathogen-mediated interference with IFN signaling and unleashes GBP1 as part of an ETI response.

In addition to binding to bacteria directly, human GBP1 and its mouse ortholog GBP2 bind to PcVs formed by pathogens such as *Legionella* and GAS [336–339], and are also dependent on GTPase activity and prenylation [338]. Bacterial secretion systems or bacterial toxins inserted into membranes mark PcVs as GBP targets [336,338]. Recruitment of human GBP1 and mouse GBP2 to PcVs is aided by the host protein LGALS3 that detects β-galactosides exposed upon vacuolar damage in these settings [336,338]. Other modifications of the PcV such as microbe-driven changes in the lipid composition may provide additional patterns recognized by GBPs. GBPs associated with PcVs exert host defense through at least two mechanisms: lysis and autophagic degradation of vacuoles [262,322]. However, the underlying molecular mechanisms are not well defined. GBP1 stimulates the TBK1-dependent phosphorylation and recruitment of SQSTM1 to GAS-containing vacuoles. Disruption of either autophagy or *GBP1* expression results in comparable increases in GAS intracellular burden, suggesting GBP1 eliminates GAS-containing vacuoles through xenophagy [338].

In macrophages infected with *Chlamydia trachomatis*, the causative agent of the sexually transmitted disease Chlamydia, GBP1 promotes the delivery of PcVs into degradative autolysosomes and activates the NLRP3 inflammasome [340]. Activation of the NLRP3 inflammasome results in IL-1β secretion and pyroptosis. The unique catalytic ability of GBP1 to process GTP into guanosine monophosphate (GMP) through two consecutive cleavage reactions [341,342] is essential for GBP1-mediated NLRP3 activation [340]. The catabolism of GBP1-generated GMP ultimately results in the production of uric acid [340], an established NLPR3 agonist. However, the functional relationship between GBP1-mediated xenophagy and NLRP3 activation is not well defined. Delivery of *C. trachomatis*-containing vacuoles into autolysosomes through GBP1 requires the first but not the second hydrolysis step [340]. Therefore, GBP1-dependent autolysosomal degradation of *C. trachomatis* can be functionally uncoupled from GBP1-dependent pyroptosis. Whether successful xenophagic removal of *C. trachomatis* and other intracellular pathogens limits GMP production by GBP1 and thereby reduces inflammation remains an unanswered question.

## Non canonical pathways of LC3 conjugation in antimicrobial defense

### CASM, LAP and LANDO

**Figure 5.**
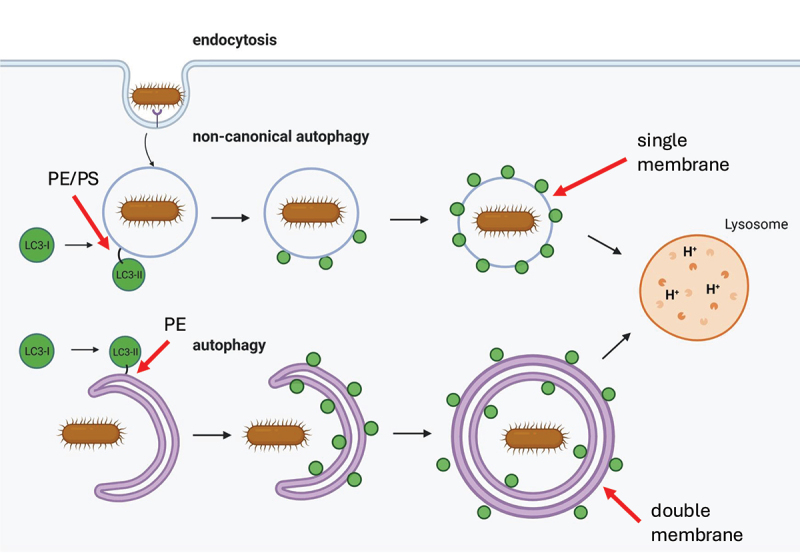
Autophagy and LAP provide different ways to control infection. Canonical autophagy conjugates LC3 to the lipid phosphatidylethanolamine (PE) to generate double-membraned autophagosomes that engulf pathogens in the cytosol. Non-canonical CASM pathways that include LC3 associated phagocytosis (LAP) conjugate LC3 to PE and phosphatidylserine (PS) on single-membraned endosomes and phagosomes containing extracellular pathogens as they enter cells. In addition to differences in membrane structures (single versus double, indicated by arrows), these pathways are distinguished by the location of the pathogen at the time of initiation. In both cases, conjugation of LC3 to vacuoles containing pathogens facilitates fusion with lysosomes leading to pathogen degradation. Therefore, by mediating both autophagy and CASM, the autophagy machinery can target both cytosolic and non-cytosolic pathogens. Figure adapted from Wang et al. [343].

Many pathogens enter cells by endocytosis or phagocytosis and are invisible to canonical autophagy pathways in the cytosol because they are protected by the endosome/phagosome membrane. This provides an opportunity for viruses to release genomes into the cell and for bacteria and parasites to generate vacuoles specialized for replication. This potential gap in microbial defense can be filled by alternative “non-canonical” autophagy pathways that repurpose a subset of ATG proteins to conjugate LC3/Atg8 to the cytosolic face of endosomes and phagosomes containing pathogens (Figure 5). This has been demonstrated for viruses, bacteria, and parasites and is the subject of a recent review [343]. The function of single versus double membrane LC3 conjugation can be considered similar in many cases because the sequestered cargo is targeted to the lysosome in either scenario. Where these pathways often differ is in the upstream trigger and the localization of the substrate at the time of initiation, i.e., cytosolic for autophagy and endosomal/extracellular for CASM.

Alternative pathways for LC3 conjugation were first suspected when studies using macrophages showed recruitment of LC3 to phagosomes containing *E. coli* or killed yeast, and when macrophages were incubated with TLR ligands [344]. LC3 conjugation required ATG5 and ATG7 but LC3 was recruited to phagosomes with single membranes rather than double-membraned autophagosomes. Parallel studies showed that LC3 conjugation to phagosomes was dependent on NADPH oxidase, a membrane-bound complex that generates ROS [345]. This process was called **LAP** (**L**C3-**a**ssociated **p**hagocytosis) to indicate a pathway occurring in phagocytes during the uptake and eventual degradation of pathogens and apoptotic cells in lysosomes. The observation that LC3 was conjugated to *Salmonella*-containing vacuoles in epithelial cells demonstrated that LC3 conjugation to single membranes was not restricted to phagocytic cells [345].

It is now clear that non-canonical conjugation of LC3 to single membranes can occur in many different cell types and can be induced by a variety of stimuli [346–349]. Examples include perturbation of osmotic balance within endocytic vesicles by lysosomotropic drugs, pore forming proteins, and stimulation of lysosome Ca2+ channel MCOLN1/TRPML1. Conjugation also occurs during uptake of particulate material such as transfection reagents, apoptotic cells, or when pancreatic acinar cells engulf exocytic zymogen granules [346–349]. The acronym **CASM** has been coined as an umbrella term to describe the **c**onjugation of **A**TG8 to **s**ingle **m**embranes [350]. Conjugation of LC3 to endosomes has been called **LANDO** to describe **L**C3 **a**ssociated e**ndo**cytosis, a pathway that can modulate endocytosis and signaling of cytokine receptors and has been implicated in the pathogenesis of Alzheimer’s disease where it enhances clearance of β-amyloid [351,352].

In phagocytic cells, LAP is mediated by a complex of proteins containing Rubicon/RUBCN, BECN1, UVRAG, PIK3R4/VPS15, and PIK3C3/VPS34 that act upstream of the core ATG12–ATG5-ATG16L1 complex. RUBCN binds to the CYBA/p22phox subunit of the NADPH oxidase complex to increase production of ROS within the phagosome. Sustained production of ROS increases the pH within the phagosome and provides a signal for assembly of the vacuolar-type ATPase (V-ATPase) and recruitment of the ATG12–ATG5-ATG16L1 complex to the phagosome to conjugate LC3 to phosphatidylethanolamine and phosphatidylserine [350,353]. ROS production may also contribute to CASM in non-phagocytic cells since diphenyliodonium (DPI), an inhibitor of CYBB/NOX2, and siRNA for *CYBA* inhibit LC3 conjugation to PcVs in epithelial cells infected with *Salmonella* [345]. Also, DPI inhibits LC3 conjugation in fibroblasts incubated with monensin or chloroquine [354]. LC3 conjugation is also inhibited by ATG4, a cysteine protease that cleaves LC3 from membranes to recycle LC3 back to the cytosol. ROS produced in response to TLR signaling inhibit ATG4 and slow removal of LC3, causing increased processing and presentation of antigens on class II MHC [355].

**Figure 6.**
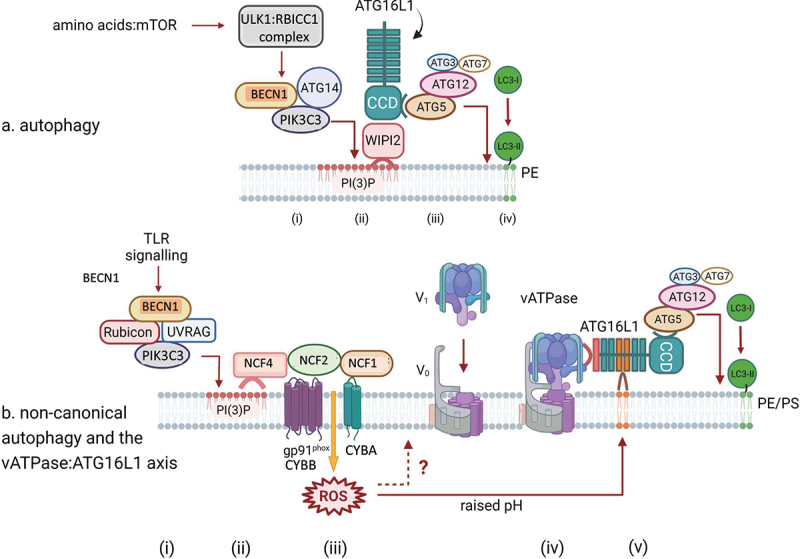
Pathways for conjugation of LC3 to membranes during autophagy and the CASM pathways that use the V-ATPase:ATG16L1 axis. (A) autophagy. Autophagy is activated in response to a fall in amino acids which leads to inhibition of mTOR and activation of the ULK1:RB1CC1 (FIP200) initiation complex (i) and downstream activation of the PtnIns3kinase complex containing BECN1 (Beclin1), ATG14 and PIK3C3 (VPS34). The PtnIns3 kinase activity of PIK3C3 generates PtdIns3P in autophagosome membranes which provide a platform for binding of WIPI2 (ii). WIPI2 binds to the coiled coil domain (CCD) of ATG16L1 leading to recruitment of the LC3 conjugation complex (ATG5-ATG12, ATG3, ATG7). The conjugation reaction converts LC3I to LC3II leading to covalent binding of LC3 to phosphatidylethanolamine (PE) in the autophagosome membrane (iii-iv). (B) LC3 conjugation via CASM and the V-ATPase:ATG16L1 axis. LC3 conjugation by CASM in phagocytic cells (LAP) is activated by TLR signaling through a complex containing RUBCN, BECN1, UVRAG, PIK3R4, and PIK3C3 (i). TLR signaling activates PIK3C3 leading to generation of PtdIns3 in phagosome membranes (ii). This generates a binding site for NCF4/p40phox which stabilizes the NADPH complex (CYBB/NOX2, NCF1/p47phox, NCF4/p40phox, NCF2/p67phox). at the same time binding of RUBCN to CYBA/p22phox increases production of reactive oxygen species (ROS). Generation of ROS increases the pH in the lumen of the phagosome (iii) stimulating assembly of the V_o_ V_1_ subunits of the V-ATPase (iv). the V-ATPase binds to the WD domain of ATG16L1 leading to recruitment of the LC3 conjugation complex (ATG12–ATG5, ATG3, ATG7) to the phagosome and conjugation of LC3 to phosphatidylserine (PS) and PE in the phagosome membrane (v). a similar ROS-dependent pathway involving assembly of the V-ATPase operates in non-phagocytic cells, but the precise components of the NADPH oxidase complex are unclear. Figure adapted from Wang et al. [343].

The site of LC3 conjugation is determined by the binding of ATG16L1 within the ATG12–ATG5-ATG16L1 complex to membranes [356]. During canonical autophagy, the N-terminal domain of ATG16L1 directs conjugation of LC3 to phagophores by binding WIPI2, which is recruited to sites enriched for PtdIns3P generated by PIK3C3 (Figure 6A). In contrast, conjugation of LC3 to single membranes during LAP in dendritic cells and CASM in epithelial cells requires the WD repeat domain at the C terminus of ATG16L1 (Figure 6B) [357,358]. This domain contains a lysine residue at position 490 that is crucial for CASM and several amino acids that bind phospholipids *in vitro* that are required for binding ATG16L1 to perturbed endosome membranes [358,359]. Mice lacking the linker and WD domain (ΔWD) of ATG16L1 [360] are defective in CASM and LAP but preserve canonical autophagy because they retain the N-terminal amino acids required to bind WIPI2. Unlike autophagy gene knockout mice, the ΔWD mice survive neonatal starvation, grow normally and maintain tissue homeostasis. Importantly for infection studies, the ΔWD mice do not have the upregulated IFN-I signaling and inflammasome activation observed in mice lacking full length ATG16L1 in macrophages [247]. Intranasal challenge has shown that ΔWD mice are highly sensitive to influenza A virus (IAV) leading to extensive lung inflammation and high mortality [354]. Cell-type-specific knockout mice using *Lyz2-Cre* combined with bone marrow transfers and radiation chimeras suggest that ATG16L1 with an intact WD domain in epithelial cells rather than phagocytic cells protects the lungs against IAV infection *in vivo*. Bone marrow derived cells from ΔWD mice have shown that the WD domain enhances cytokine responses of dendritic cells to commensal yeast [357] and facilitate antigen presentation [358].

### SopF and the V-ATPase-ATG16L1 axis (VAIL)

Research into bacterial recognition by host autophagy pathways has focused on the recruitment of LC3 around bacteria to restrict their proliferation. The model organism *Salmonella* has been extensively studied in this context [22]. However, the relatively low level of recruitment of LC3 to approximately 10% of vacuoles has hindered progress [22]. A *Salmonella* transposon screen revealed that disruption of the T3SS effector SopF dramatically increases the numbers of bacteria trapped in LC3-positive vacuoles [84]. SopF impedes LC3 translocation induced by various bacterial infections without affecting canonical autophagy, indicating that SopF inhibits a key step in the recruitment of LC3 to vacuoles containing bacteria by CASM.

Further investigations identified V-ATPase as a pivotal component in this process from two independent perspectives. First, a host genome-wide CRISPR/Cas9 screen demonstrated that disruption of the V-ATPase blocks bacteria-induced LC3 lipidation. Second, mechanistic studies revealed that SopF catalyzes the ADP-ribosylation of Voc, the c-ring of V-ATPase, at Gln124 in a reaction that is facilitated by host cell ARF GTPases [84,361]. Beyond its role as a proton pump, the V-ATPase localized on bacteria-containing vacuoles contributes to LC3 recruitment through direct interaction with ATG16L1, primarily mediated by the C-terminal WD40 domain. SopF modification or mutation of the Gln124 site of Voc completely disrupts the binding between V-ATPase and ATG16L1, while its proton translocation function remains unaffected [84]. This critical interaction recruits the ATG12–ATG5-ATG16L1 complex to the PcV, thereby facilitating LC3/GABARAP (mammalian Atg8-family proteins) lipidation. This novel pathway has been called the V-ATPase-ATG16L1 axis or **VAIL** for **V**-ATPase-**A**TG16L1**-i**nduced **LC3** lipidation and represents the best characterized pathway for conjugation of LC3/ATG8s to single membranes during CASM.

The integral membrane subunits of the V_0_ domain anchor the V-ATPase to the endolysosome membrane and generate a pore to transport protons into the lumen of the vacuole. The V-ATPase activity required for proton transport is provided by the subunits of the V1 domain which are recruited from the cytosol. Damage to PcVs through membrane insertion of bacterial secretion systems or pore-forming toxins allows leakage of H+ ions to slow V-ATPase-mediated acidification of vacuoles. It has been proposed that V-ATPase can sense such changes in vacuolar pH [362], which provides a signal for recruitment of the V_1_ subunits from the cytosol to the membrane bound V_0_ subunits [353]. In this way, assembly of the V-ATPase provides a binding site for ATG16L1 which recruits the LC3 conjugation machinery (ATG12–ATG5-ATG16L1) onto vacuoles to activate CASM or VAIL. This has been confirmed by a CRISPR-Cas9 screen showing that recruitment of LC3 to the vacuole induced by the proton channel generated by the influenza A virus M2 protein required the V-ATPase and that binding of ATG16L1 could be inhibited by SopF [363]. A more recent study demonstrated the direct binding of ATG16L1 to amino acids 176–191 in N-terminal domain of the ATP6V1H subunit of the V-ATPase which forms a loop at the interface between the V_1_ and V_0_ complexes. Interestingly, a short isoform of ATP6V1H, principally expressed in neuronal cells, lacks this loop and is unable to bind ATG16L1, suggesting that CASM may be less active in neurons [364]. ATG16L1 can also be recruited to endocytic compartments by over expression of TMEM59, a type 1 membrane glycoprotein found in late endosomes and lysosomes [365,366]. The cytoplasmic domain of TMEM59 contains a short 19 amino acid domain that binds directly to the WD domain of ATG16L1 and is required for recruitment of LC3 to endosomes containing *S. aureus.*

### The V-ATPase-ATG16L1 axis is activated during STING1 signaling

cGAMP generated by the DNA sensor cGAS activates STING1 to induce IFN-I signaling, inflammasome activation, and the formation of LC3 puncta (marker of LC3 membrane association) [367,368]. C-terminal residues of STING required for IFN induction are not required for LC3 conjugation, suggesting that pathways leading to LC3 conjugation are separate. STING is located to the ER by a multi-spanning transmembrane domain. Activation of STING by cGAMP induces a conformational change that releases STING1 from the ER allowing transport to the Golgi in COPII coated vesicles [368]. The vesicles have a single membrane and recruit the ATG5-ATG12:ATG16L1 complex to conjugate LC3 independently of the ULK1/ULK2 and RB1CC1 initiation complex, suggesting recruitment through CASM [369]. Further in common with CASM, activation of STING1 leads to recruitment of the V_1_ complex of the V-ATPase from the cytosol to the perinuclear Golgi vesicles containing LC3 by a pathway dependent on the WD domain of ATG16L1 [369]. A connection with raised pH within the secretory pathway is provided by the observation that STING increases the pH of the cis and medial Golgi stacks and that the multi-spanning transmembrane domain of STING generates a proton translocating channel when reconstituted in proteoliposomes [370]. Furthermore, conjugation of LC3 to membranes is inhibited when cells are incubated with C53, a STING1 agonist that binds to the transmembrane domain, implicating the proton channel.

STING1-mediated conjugation of LC3 to membranes can be demonstrated in *Nematostella vectensis*, a small sea anemone that evolved 500 million years ago. The *N. vectensis* STING1 protein lacks the C-terminal domain required for IFN induction. Similarly, STING1 from *Xenopus tropicalis* also lacks the C terminus required for TBK1 and IRF3 activation but can conjugate LC3 to single membranes [368]. It is possible that the ability of STING1 to drive LC3 conjugation to membranes is very ancient and evolved before the emergence of IFN-I pathways in vertebrates. Furthermore, the proton channel activity of STING1 is required for activation of the inflammasome in response to cytosolic DNA, but inflammasome activation is uncoupled from LC3 conjugation because it is independent of ATG16L1390. Precisely how recruitment of LC3 to Golgi-derived vesicles by STING1 controls infection has not been resolved. The perinuclear vesicles positive for LC3 induced by STING1 are reported to capture DNA and DNA viruses from the cytosol [368] and could therefore engulf microbes in the cytoplasm through either autophagy or another process. LC3-positive vesicles containing STING travel to lysosomes [368]. Rather than degrading pathogens, it is possible that LC3 conjugation is used to downregulate inflammasome and IFN signaling by delivering STING1 to lysosomes for degradation.

### Cooperation between autophagy and CASM

Autophagosome formation and single-membrane LC3 targeting compete for similar sets of ATG proteins and may not occur at the same time, but sequential action is possible. Following the hijacking of the polymeric IgA receptor (pIgR), *S. pneumoniae* becomes trapped within endosomes of human nasopharyngeal epithelial cells. The lysis of a proportion of these bacteria releases a pore-forming toxin, pneumolysin (Ply), to inhibit endosome acidification [371]. Without Ply, *S. pneumoniae* are killed within 1 h by the endocytic pathway. Ply activity is fine-tuned by pneumococcal sialidase NanA, which trims the sialylated glycans of endosomal or endo-lysosomal membrane proteins to suppress Ply binding and promote the intracellular survival of pneumococci [372]. This fine-tuning is necessary because Ply acts as a double-edged sword, with subsequent endosomal damage inducing the formation of Pneumococci-containing LAPosome-like vesicles (PcLVs) and Pneumococci-containing autophagic vesicles (PcAVs) [373]. PcLVs display LC3 on single membranes are transiently induced after 1 h of infection and are dependent on several autophagy-related factors including SQSTM1, CALCOCO2, ATG5, and C-terminal WD domain of ATG16L1 (Figure 2) [373]. Although PcLV formation is not directly involved in bactericidal processes, it is a prerequisite for the formation of PcAVs representing RB1CC1- and PtdIns3P-dependent autophagosomes, which form around 2 h after invasion [373]. For this to occur, LC3B and CALCOCO2 first must disappear from the periphery of the pneumococci-containing vesicles, with SQSTM1 remaining. Then LC3B is recruited back to *S. pneumoniae* with autophagosomes consisting of a canonical LC3B-positive bilayer structure that, upon fusion with lysosomes, is bactericidal. The induction and maintenance of these autophagic vacuoles requires RAB41, a Rab family member protein involved in intracellular membrane trafficking that is localized in the Golgi apparatus. In addition, K63-linked ubiquitin chains formed by NEDD4/Nedd4–1 are required, whereas other xenophagy-associated E3 ligases such as LRSAM1, TRIM16, and SMURF1 are absent from the PcAV [371]. During this process, LC3B and GABARAPL1 are recruited to both the PcLV and PcAV, whereas LC3A, GABARAP, and GABARAPL2 are specific to the earlier forming PcLV. Then, although LC3A and GABARAPL1 are required for CASM induction, it is GABARAPL2 and GABARAP that regulate CASM shedding and subsequent induction of xenophagy, respectively. In this manner, different LC3/GABARAP proteins coordinate the progression of CASM to xenophagy (Figure 2) [374].

A similar sequence of events occurs during *Listeria* infection of macrophages. LC3 initially decorates single membrane phagosomes upon activation of the microbe-binding integrin Mac-1 [375]. When this pathway fails to kill *Listeria*, the bactericidal activity occurs subsequently when the bacterium accesses the cytosol and triggers xenophagy [376]. This activity of xenophagy only occurs when bacterial phospholipases and ActA are inactivated and *Listeria* cannot evade the pathway. LLO from *Listeria* can induce an additional form of CASM that is independent of the NADPH oxidase, although this pathway is insufficient to kill the bacteria [377]. LC3 can also be conjugated to the single membrane vacuole that encloses *Salmonella*. In this case, TECPR1 binds cytosol exposed sphingomyelin on ruptured vacuoles to recruit ATG5 with LC3 conjugated to the membrane in an ATG16L1-independent manner [378,379]. These studies highlight the importance of studying both CASM and classical xenophagy upon the perturbation of different pathogen-containing vacuoles.

## Autophagy and control of extracellular bacteria

While autophagy is essentially an intracellular process, it has far-reaching effects beyond the boundaries of the cell. These extracellular effects are most pronounced in mucosal tissues, where the host must maintain proper separation between its own tissues and the environment, consistent with the genetic link between autophagy and Crohn disease. This separation prevents attachment of commensal microbes to host tissue and invasion of pathogens. To provide this separation, the host secretes a variety of proteins that together with epithelial cells comprise the mucosal barrier [380]. Here, we build on the prior sections that define how autophagy controls internalized microbes by discussing the roles autophagy plays in maintaining this barrier through control of the extracellular environment.

### Autophagy in epithelial cells dictates host-microbe interaction in mucosal tissues

**Figure 7.**
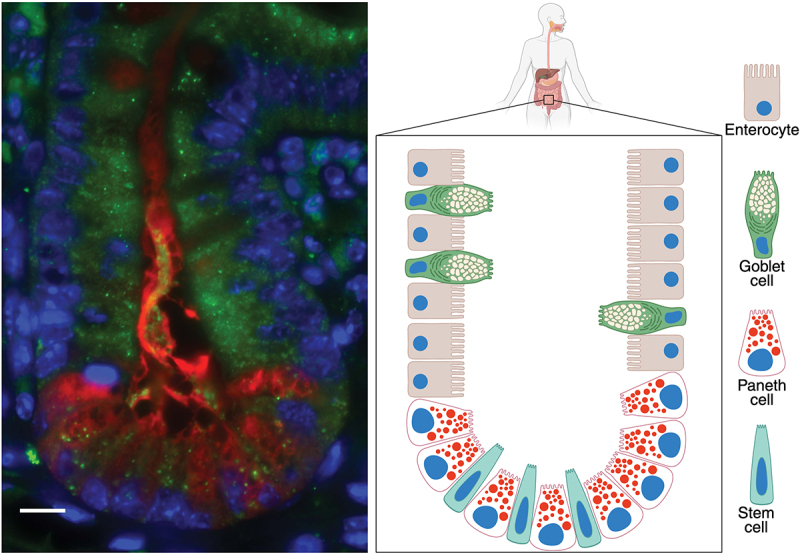
Secretory epithelial cells that protect the intestinal barrier are dependent on autophagy. Left panel: fluorescent microscopy of a mouse small-intestinal crypt with LYZ (lysozyme) labeled in red, LC3 in green, and DNA in blue. LYZ is packaged in granules along with other antimicrobial proteins and can be seen secreted from Paneth cells at the crypt bottom into the lumen. Autophagosomes can be seen as green puncta. This process is disrupted upon inhibition of autophagy, such as through mutation of the Crohn disease gene *ATG16L1*. Image contributed by Shai Bel, Bar-Ilan University. Right panel: cartoon rendering of a small intestinal crypt showing the positioning of Paneth cells relative to epithelial stem cells, goblet cells, and enterocytes. Through their antimicrobial properties, Paneth cells protect the crypt and the epithelial stem cell niche. They work together with the mucus-secreting goblet cells and other immune mechanisms to establish a chemical barrier against invasive microbes.

Epithelial cells in mucosal tissues secrete mucus and antimicrobial proteins, which together form chemical barriers that protect the host from bacterial encroachment and facilitate proper symbiosis with commensal microbes [381]. In the intestine, the cells charged with secreting mucus and antimicrobial proteins are goblet and Paneth cells, respectively [380] (Figure 7). As professional secretory cells, goblet and Paneth cells must maintain high translation and secretion levels to maintain the mucosal barrier. This high demand for protein synthesis requires the ER to constantly synthesize, fold, and properly package new proteins for secretion [382]. Therefore, goblet and Paneth cells are especially sensitive to disruption of the unfolded protein response (UPR) pathway. Genetic deletion in key components of the UPR in mice leads to accumulation of ER stress and consequent dysfunction of goblet and Paneth cells. The physiological results of this dysfunction, at the organism level, are breakdown of the mucosal barrier, infiltration of bacteria into the intestinal tissue, and finally development of chronic gut inflammation [383].

A hallmark of IBD is dysfunction of the mucosal barrier. Studies in transgenic mice found that mutations in autophagy genes, including carriage of the ATG16L1T300A variant, result in dysfunction of goblet and Paneth cells [384–388], dysbiosis of the gut microbiota [389], and higher susceptibility to intestinal inflammation [220,388,390]. These dysfunctions were also confirmed in humans carrying these mutations [391–394]. In cultured cells, UPR leads to activation of autophagy, which prevents accumulation of ER stress [395]. A landmark study in mice showed that simultaneous loss of the UPR gene *Xbp1* (X-box binding protein 1) and *Atg16l1* within Paneth cells induces intestinal inflammation resembling IBD in humans [396]. A follow-up study has further shown how Paneth cell dysfunction in secretion of antimicrobial proteins in these mice alters the gut microbiota [397]. The dependence of the UPR on autophagy to relieve ER stress was also confirmed in intestinal goblet cells in which constitutive activation of autophagy via a mutation in *Becn1* leads to reduction of ER stress and subsequent excess secretion of mucus in the intestine. This increase in mucus secretion reshaped the gut microbiome, with enrichment of mucus-degrading bacteria, and protection from a mouse model of IBD [398]. Thus, the autophagy process can orchestrate immunity in the gut and control of extracellular bacteria residing in the gut lumen by alleviating ER stress in intestinal secretory cells.

Consistent with a role for microbes in Crohn disease, autophagy is necessary to buffer microbial triggers of epithelial cell defects. A norovirus strain that induces a beneficial cytokine response in other settings triggers Paneth cell functional defects and cell death in *Atg16L1* mutant mice by interfering with anti-inflammatory γδ T cells [399–403]. *Salmonella* can also inhibit this anti-inflammatory property of γδ T cells [403], suggesting that the identity of the infectious trigger is less important than the quality of the host response in the context of autophagy deficiency. Human intestinal Paneth cells with combined risk variants in both *NOD2* and *ATG16L1* demonstrate even more pronounced morphological alterations [392], demonstrating cooperation between genetic risk pathways.

Autophagy in intestinal epithelial cells affects the gut microbiota and intestinal homeostasis in various additional ways. Autophagy in intestinal epithelial cells leads to accumulation of Immunoglobulin A (IgA)-producing plasma cells in the intestinal tissue of mice, a finding confirmed in humans [404]. As the most abundant immunoglobulin secreted in mucosal tissues, IgA antibodies shape the gut microbiota and intestinal immunity. The autophagy machinery can also be used to secrete, not only degrade. When invasive bacteria disrupt the ability of Paneth cells to secrete the antimicrobial enzyme lysozyme via the Golgi, these cells can reroute their cargo to secretion via autophagosomes [384]. The secretion of mucin granules by goblet cells also requires autophagy proteins that are associated with NADPH oxidase activity [405]. This use of autophagy as an alternative secretion mechanism, sometimes referred to as secretory autophagy, can apply to exocytosis of proteins lacking a secretion signal when the autophagosome or amphisome fuses with the plasma membrane instead of the lysosome (e.g., IL-1B when it is incorporated in-between the two membranes of the autophagosome) or through less understood ATG-dependent processes [1,406–409]. Mice carrying the IBD-associated T300A mutation in ATG16L1 were unable to activate secretory autophagy in Paneth cells [410]. Thus, autophagy in epithelial cells can affect gut bacteria and host immunity in multiple, distinct ways.

### Autophagy-AIEC interactions

The role of autophagy in host-microbiota interaction is not specific to a single microbe [411]. However, some bacterial strains are more strongly associated with IBDs and examination of how these bacteria intersect autophagy has informed mechanism. More than 25 years ago, a new pathovar of *E. coli*, designated as AIEC (adherent-invasive *E. coli*), was identified with higher prevalence in patients with Crohn disease compared to healthy individuals [412,413]. The inflamed gut environment increases the availability of oxygen and nitrates as electron acceptors for microbial respiration, leading to overexpansion of Enterobacteriaceae, such as *E. coli* and *Klebsiella* species, that can sustain or worsen disease through their virulence strategies [414]. AIEC bacteria were originally defined as *E. coli* strains that are able to adhere to and invade intestinal epithelial cells (IECs). This strategy enables these strains to survive and replicate within macrophages and induce pro-inflammatory cytokine production [412]. Numerous studies have identified the virulence factors of AIEC, the impact of infection on intestinal homeostasis, gut microbiota, and immune responses [415,416].

Among these are the studies on the role of autophagy in host defense to AIEC infection, which exemplifies how autophagy can respond to and control extracellular bacteria that have acquired virulence strategies that promote invasion and breach of barrier, a key process involved in Crohn disease etiology [417,418]. Upon AIEC infection, autophagy is induced in IECs, and a functional autophagy is necessary to limit the intracellular replication of AIEC [222]. In macrophages, AIEC infection induces the recruitment of the autophagy machinery at the site of phagocytosis to limit bacterial replication and secretion of pro-inflammatory cytokines [419]. Autophagy induction upon AIEC infection in IECs requires activation of the metabolic stress response EIF2AK4-EIF2A-ATF4 pathway, which leads to the binding of the transcription factor ATF4 to the promoters of several autophagy-related genes [420]. Furthermore, mouse models with *Eif2ak4* deficiency exhibit autophagy defects and a chronic persistence of AIEC [421]. In these mice, an alteration in the gut microbiota composition precedes the appearance of intestinal inflammation. This highlights the effect of a combination between genetic factors that lead to dysfunctional autophagy, gut dysbiosis and abnormal colonization of AIEC in the initiation of intestinal inflammation [421]. The AIEC LF82 reference strain, isolated from a chronic ileal lesion of a patient with Crohn disease [413], possesses a pathogenicity island similar to the high pathogenicity island of *Yersinia spp* [422], which encodes the yersiniabactin siderophore required for iron uptake and bacterial growth [423]. It was shown that yersiniabactin is necessary for LF82-induced expression of HIF-1α, which is implicated in autophagy activation in infected IECs [424]. Thus, yersiniabactin, which is an advantage for AIEC to grow in a competitive environment, could be a disadvantage for the bacteria as it activates autophagy, leading to bacterial clearance [424].

AIEC can inhibit autophagy activation in IECs by up-regulating microRNAs 130a and 30c, which negatively regulate the expression of *ATG16L1* and *ATG5*, respectively [425]. This in turn leads to increases in AIEC intracellular replication and AIEC-induced inflammation *in vitro* and *in vivo* [425]. Furthermore, upon AIEC infection, host cells secrete exosomes [426], extracellular vesicles of 30–100 nm, which can transfer *MIR30C* and *MR130A* from cell-to-cell to inhibit autophagy, favoring AIEC intracellular replication [427]. AIEC can also inhibit autophagy by impairing host SUMOylation [428], an eukaryotic-reversible post-translational modification, in which SUMO, a ubiquitin-like polypeptide, is covalently linked to target proteins. Overexpression of SUMO in IECs enhances autophagy, inhibiting AIEC intracellular replication [428].

Crohn disease-associated variants in *ATG16L1*, *IRGM*, and *NOD2* are linked with a defect in autophagy-mediated AIEC clearance accompanied by enhanced pro-inflammatory cytokine production in epithelial cells and macrophages [222,419,429]. The blood monocyte-derived macrophages (BMDMs) from patients with Crohn disease exhibit increased AIEC replication compared to those from healthy individuals [430], and this defect in AIEC clearance is linked to the Crohn disease-associated variants in the autophagy-related genes *IRGM* and *ULK1* [431]. Furthermore, perturbation of the autophagy flux upon AIEC infection has been reported in blood monocyte-derived dendritic cells from Crohn disease patients expressing the ATG16L1T300A risk variant [432]. Recently, SQTM1 and CALCOCO2 were identified as xenophagy receptors for AIEC in IECs [433]. The Crohn disease-associated variant in the CALCOCO2-coding region is correlated with increased replication of the AIEC LF82 strain in BMDMs from patients with Crohn disease (unpublished data from HTTN). This was not observed for BMDMs from healthy subjects or patients with ulcerative colitis, another subtype of IBD. Autophagy is also required for a proper adaptive immune response to AIEC infection. Infection of dendritic cells by AIEC activates autophagy, contributing to the elimination of intracellular AIEC and to the activation of MHC class II antigen-specific CD4+ T cell response [201]. Furthermore, dendritic cells from patients expressing the Crohn disease-associated *NOD2* variant exhibited a reduction in lysosomal localization of AIEC bacteria, which was accompanied with diminished autophagy-mediated bacterial killing [201]. Together, these studies suggest that the Crohn disease-associated variants in autophagy-associated genes could lead to a defect in AIEC clearance by host cells, resulting in dysregulated immune responses and enhanced inflammation.

Experimental infection of mice with *Citrobacter rodentium*, a model pathogen used to study pathogenic *E. coli* infection of the gut, support a role for autophagy in controlling extracellular immunity. Whole body mutation or deletion of autophagy genes including *ATG16L1* in the intestinal epithelium leads to a spontaneous interferon signature in the colon that is dependent on exaggerating sensing of the microbiota. This increase in cytokine activity mediates an enhanced monocyte response that protects against *C. rodentium* [434–436]. In addition to defects in mitophagy that can lead to exaggerated cytokine responses to bacteria, failure to target the TLR adaptor molecule TICAM1 through SQSTM1 and TAX1BP1 in myeloid cell deficient in ATG16L1 can also increase IFN production, which protects against *Salmonella* [437]. The intensity and quality of the immune response, and whether the pathogenic bacterium encodes virulence factors that interact with ATG proteins, may determine the optimal levels of autophagy for a given microbe and cell type.

Several additional research gaps remain to be addressed to better understand the interaction between autophagy, pathogenic *E. coli* such as AIEC, and Crohn disease. Firstly, little is known about the virulence factors of AIEC that allow escape from autophagy recognition or interfere with downstream xenophagy. Secondly, the impact of Crohn disease-associated variants in autophagy genes on gut microbe control in patients remains largely unknown because autophagy is a dynamic process that is difficult to capture through gene expression or marker protein analyses. Finally, since Crohn disease is a complex disease with multifactorial etiology, it is important to consider other risk factors, such as environmental factors (*e.g*., western diet), while studying the role of autophagy genes on susceptibility to infection and its inflammatory outcomes.

### Autophagy and extracellular vesicles

Autophagy proteins mediate the production of exosomes [438–440], small endosomally derived extracellular vesicles, which have a central role in host defense. The mechanism by which exosome biogenesis intersects autophagy remains an active area of research and may involve a role for the LC3 conjugation machinery in the intraluminal budding of the multivesicular body where these intraluminal vesicles become exosomes upon fusion with the plasma membrane. Alternatively, it is possible that the role of autophagy is to generate an amphisome (autophagosome fused with the multivesicular body) with a secretory fate instead of acidification. Mice with mutations in *Atg16L1* are susceptible to *S. aurues* in models of lung and bloodstream infection due to exacerbated tissue damage caused by a bacterial pore forming toxin called α-toxin [441]. Autophagy proteins mediate the release of defensive exosomes, or defensosomes, which serve as decoys to bind and inhibit α-toxin. These defensosomes incorporate the toxin receptor ADAM10 and are induced by bacterial DNA sensing through TLR9, representing a novel host defense mechanism independent of canonical degradative autophagy [440]. Similarly, SARS-CoV-2 infection and viral RNA that activate TLRs induce ACE2-bearing defensosomes in an ATG16L1-dependent manner [442]. These vesicles bind viral particles, inhibiting infection by acting as competitive inhibitors [442,443]. This dual function of autophagy – supporting both intracellular pathogen clearance and extracellular immune evasion – highlights its versatility in host defense.

## Conclusion and outlook

Autophagy is a cornerstone of cell-autonomous immunity, playing dual roles in microbial clearance and inflammation regulation. This evolutionarily conserved pathway that maintains cellular homeostasis during nutrient deprivation or organelle stress is repurposed by host cells to target intracellular pathogens via xenophagy and CASM, while pathogens, in turn, deploy sophisticated mechanisms to evade or exploit autophagy for survival. The interplay between autophagy and bacterial infections extends beyond pathogen degradation, influencing immune signaling, tissue homeostasis, and even extracellular defense through mechanisms like secretory autophagy and defensosome release. When taking this holistic view, autophagy proteins can be viewed as molecular machines that mediate related membrane trafficking processes at the host-microbe interface inside and outside of cells. As such, their role in host defense can conform to the virulence strategies of the specific pathogen and the affected cell types and organs, thereby participating in immunity to a broad array of microbes.

Therapeutic modulation of autophagy holds promise for diseases linked to autophagy dysfunction, such as Crohn disease and chronic infections [444]. Enhancing autophagy could mitigate inflammation in IBD by reducing inflammasome activation and bacterial burden or restoring the function of secretory IECs. Conversely, autophagy inhibition – while detrimental in some contexts – might paradoxically serve as a form of ETI, where pathogens that disrupt autophagy inadvertently trigger inflammatory responses to mobilize broader immune defenses. This duality underscores the need for cell- and context-specific strategies to balance autophagy’s pro-survival and immunoregulatory functions.

A major challenge lies in disentangling the roles of canonical autophagy from pathways like LAP and CASM. Although these pathways share molecular machinery and both result in trafficking of cargo to the lysosome, their purpose may differ because of the timing of their activation and the localization of the substrate (e.g., the stage of the pathogen’s life cycle). For certain pathogens, targeting single-membrane compartments potentially prioritizes antigen presentation or inflammation modulation over degradation. Additionally, it remains unclear to what extent the various forms of single-membrane LC3/Atg8 targeting differ from one another. Understanding these distinctions and identifying which bacteria are vulnerable to defense pathways regulated by autophagy proteins can facilitate the design of antimicrobial and anti-inflammatory therapies that promote the desired responses without affecting the entire network of inter-related membrane trafficking events.

## Glossary

ACD11: Accelerated Cell Death 11; a protein involved in autophagy-dependent cell death.

ACE2: angiotensin converting enzyme 2; a receptor for SARS-CoV-2.

ADAM10: ADAM metallopeptidase domain 10; a receptor for bacterial toxins like α-toxin.

AIEC: Adherent-invasive *Escherichia coli*; a pathovar associated with Crohn disease.

AKT/Protein kinase B: AKT serine/threonine kinase; involved in cell signaling pathways.

AMPK: AMP-activated protein kinase; a cellular energy sensor that activates autophagy.

ARF: ARF GTPase; a GTPase involved in vesicle trafficking.

ARIH1: riadne RBR E3 ubiquitin protein ligase 1’; involved in ubiquitin-dependent xenophagy.

ATG: autophagy related; proteins involved in the autophagy pathway (e.g., ATG5, ATG7, ATG12, ATG16L1).

ATF4: activating transcription factor 4; involved in stress responses and autophagy regulation.

BAK1: Brassinosteroid-associated kinase 1; a PRR co-receptor in plants.

BI-1: Bax inhibitor 1; involved in ER stress and autophagy.

BMDMs: blood monocyte-derived macrophages.

BPA: Binding partner of ACD11; involved in autophagy regulation.

C53: A STING1 agonist that blocks proton channel activity.

CALCOCO2/NDP52: calcium binding and coiled-coil domain 2; an autophagy receptor for xenophagy.

CASM: Conjugation of ATG8 to single membranes; a non-canonical autophagy pathway.

CASP4: caspase 4; an inflammatory caspase activated by GBP1 during bacterial infection.

CbpC: *Streptococcus pneumoniae* surface protein that induces selective autophagy.

cGAMP: Cyclic GMP-AMP; a second messenger produced by CGAS.

CGAS: Cyclic GMP-AMP synthase; a cytosolic DNA sensor.

COPII: Coat protein complex II; involved in vesicle transport from the ER.

Crohn disease: A subtype of inflammatory bowel disease (IBD) linked to autophagy dysfunction.

CYBB/NOX2: cytochrome b-245 beta chain; generates ROS in phagocytes.

DAMPs: Danger/damage-associated molecular patterns; host-derived molecules that trigger immune responses.

DC: Dendritic cells.

Defensosomes: ATG16L1-dependent exosomes that act as decoys for bacterial toxins.

DPI: Diphenyliodonium; an inhibitor of NADPH oxidase.

EIF2AK4: eukaryotic translation initiation factor 2 alpha kinase 4; involved in stress responses.

ER: Endoplasmic reticulum; a site of autophagy initiation.

ESCRT: Endosomal sorting complex required for transport; involved in membrane repair.

ESX-1: Type VII secretion system of *Mycobacterium tuberculosis*.

ETI: Effector-triggered immunity; a plant immune response to pathogen effectors.

FLS2: Flagellin-sensing 2; a PRR in plants.

GABARAP: GABA type A receptor-associated protein; an Atg8 homolog.

GAPCs: Cytosolic glyceraldehyde-3-phosphate dehydrogenases; negative regulators of autophagy.

GBP: guanylate binding protein; IFN-inducible GTPases involved in host defense.

GEF: Guanine nucleotide exchange factor; activates small GTPases like RAB1.

GTPase: Enzymes that hydrolyze GTP; involved in signaling and membrane trafficking.

HIF1A/HIF-1α: hypoxia-inducible factor 1 subunit alpha; regulates autophagy under hypoxia.

HOPS: Homotypic fusion and protein sorting; a complex that mediates autophagosome-lysosome fusion.

HR: Hypersensitive response; a plant immune response involving programmed cell death.

IAV: Influenza A virus.

IBD: Inflammatory bowel disease; includes Crohn disease and ulcerative colitis.

IFN-I: Type I interferons; cytokines with antiviral and antibacterial roles.

IFNAR: interferon alpha and beta receptor; mediates IFN-I signaling.

IgA: Immunoglobulin A; an antibody class important in mucosal immunity.

IECs: Intestinal epithelial cells.

IL1B/IL-1β: Interleukin 1 beta; a proinflammatory cytokine.

IRF3: interferon regulatory factor 3; mediates IFN-I production.

IRGM: immunity related GTPase M; involved in autophagy and mitophagy.

LAMP1: lysosomal associated membrane protein 1; marks lysosomes and endosomes.

LANDO: LC3-associated endocytosis; a non-canonical autophagy pathway.

LAP: LC3-associated phagocytosis; a pathway for LC3 conjugation to phagosomes.

LLO: Listeriolysin O; a pore-forming toxin from *Listeria monocytogenes*.

LPS: Lipopolysaccharide; a component of Gram-negative bacterial cell walls.

LRSAM1: leucine rich repeat and sterile alpha motif containing 1; an E3 ubiquitin ligase.

LUBAC: Linear ubiquitin chain assembly complex; generates M1-linked ubiquitin chains.

MAP1: C3/LC3: microtubule associated protein 1 light chain 3; an Atg8 homolog.

MAPK: mitogen-activated protein kinase; involved in signaling pathways.

MCOLN1/TRPML1: mucolipin TRP cation channel 1; a lysosomal calcium channel.

MDP: Muramyl dipeptide; a bacterial cell wall component that activates NOD2.

MHC: Major histocompatibility complex; presents antigens to T cells.

Mtb: *Mycobacterium tuberculosis*.

MTMR3: myotubularin related protein 3; an autophagy inhibitor.

MTF1: metal regulatory transcription factor 1; regulates autophagy via zinc.

MTORC1: mechanistic target of rapamycin complex 1; inhibits autophagy under nutrient-rich conditions.

NADPH oxidase: An enzyme complex that generates ROS, involved in LAP.

NBR1: NBR1 autophagy cargo receptor; a selective autophagy receptor.

NCKAP1L/HEM1: NCK associated protein 1 like; involved in ER-associated condensates.

NLRs: NOD-like receptors; cytosolic PRRs in animals.

NOD2: nucleotide binding oligomerization domain containing 2; a cytosolic innate immune signaling molecule activated by MDP.

NPR1: Nonexpressor of pathogenesis-related proteins 1; a regulator of plant immunity.

OPTN: optineurin; an autophagy receptor involved in xenophagy.

ORM1: orosomucoid 1; cargo receptor for FLS2 degradation in plants.

PAMPs: Pathogen-associated molecular patterns; microbial molecules sensed by PRRs.

Paneth cells: Intestinal secretory cells that produce antimicrobial peptides.

PcV: Pathogen-containing vacuole.

PE_PGRS: Proline-glutamic acid polymorphic GC-rich sequences; *Mtb* virulence factors.

Ply: Pneumolysin; a *Streptococcus pneumoniae* toxin.

PPARA/PPAR-α: peroxisome proliferator activated receptor alpha; regulates peroxisome biogenesis.

PRRs: Pattern recognition receptors; detect microbial molecules.

PtdIns3P: Phosphatidylinositol 3-phosphate; a lipid marker for autophagosome formation.

PTI: Pattern-triggered immunity; a plant immune response to PAMPs.

PtdIns3K: Phosphatidylinositol 3-kinase; involved in autophagy initiation.

RAB7: RAB7, member RAS oncogene family; a GTPase involved in autophagosome-lysosome fusion.

RB1CC1/FIP200: RB inducible coiled-coil 1; part of the ULK1 complex.

RIPK2: receptor interacting serine/threonine kinase 2; mediates NOD2 signaling.

RIPosomes: RIPK2 oligomers formed during NOD2 signaling.

ROS: Reactive oxygen species; signaling chemicals in autophagy and immunity.

RUBCN: rubicon autophagy regulator; regulates LAP.

SAR: Systemic acquired resistance; a plant immune response.

SFN/14–3–3σ: stratifin; a protein that sequesters phosphorylated GBP1 to prevent proinflammatory cell death.

SINCs: SA-induced NPR1 condensates, involved in plant immunity.

SQSTM1/p62: sequestosome 1; an autophagy receptor for ubiquitinated cargo.

STING1: stimulator of interferon response cGAMP interactor 1; activates IFN-I responses to cytosolic DNA.

TBK1: TANK binding kinase 1; phosphorylates autophagy receptors.

TICAM1/TRIF: TIR domain containing adaptor molecule 1; a TLR adaptor.

TLRs: Toll-like receptors; PRRs sensing microbial molecules.

TMEM59: transmembrane protein 59; a membrane glycoprotein involved in CASM.

TOR: target of rapamycin; a kinase that inhibits autophagy.

T3SS: Type 3 secretion system; an apparatus used by bacterial to secrete virulence factors

UPR: Unfolded protein response; alleviates ER stress.

V-ATPase: Vacuolar-type ATPase; acidifies organelles and regulates CASM.

VAIL: V-ATPase-ATG16L1-induced LC3 lipidation; a CASM pathway.

VAMP8: vesicle associated membrane protein 8; mediates autophagosome-lysosome fusion.

VPE: Vacuolar processing enzyme; involved in plant cell death.

WIPI2: WD repeat domain, phosphoinositide interacting 2; recruits ATG16L1.

XBP1: X-box binding protein 1; a transcription factor in the UPR.

Xenophagy: Selective autophagy targeting intracellular pathogens.

Zymogen granules: Secretory vesicles in pancreatic acinar cells.

## Data Availability

Data sharing is not applicable to this article as no data were created or analyzed in this study.
